# *In silico *analysis of expressed sequence tags from *Trichostrongylus vitrinus *(Nematoda): comparison of the automated ESTExplorer workflow platform with conventional database searches

**DOI:** 10.1186/1471-2105-9-S1-S10

**Published:** 2008-02-13

**Authors:** Shivashankar H Nagaraj, Robin B Gasser, Alasdair J Nisbet, Shoba Ranganathan

**Affiliations:** 1 Department of Chemistry and Biomolecular Sciences, Macquarie University, Sydney, NSW 2109, Australia; 2 Department of Veterinary Science, The University of Melbourne, Werribee, VIC 3030, Australia; 3 Parasitology Division, Moredun Research Institute, Pentlands Science Park, Bush Loan, Penicuik, EH26 0PZ, UK; 4 Department of Biochemistry, Yong Loo Lin School of Medicine, National University of Singapore, Singapore 119260

## Abstract

**Background:**

The analysis of expressed sequence tags (EST) offers a rapid and cost effective approach to elucidate the transcriptome of an organism, but requires several computational methods for assembly and annotation. Researchers frequently analyse each step manually, which is laborious and time consuming. We have recently developed ESTExplorer, a semi-automated computational workflow system, in order to achieve the rapid analysis of EST datasets. In this study, we evaluated EST data analysis for the parasitic nematode *Trichostrongylus vitrinus *(order Strongylida) using ESTExplorer, compared with database matching alone.

**Results:**

We functionally annotated 1776 ESTs obtained *via *suppressive-subtractive hybridisation from *T. vitrinus*, an important parasitic trichostrongylid of small ruminants. Cluster and comparative genomic analyses of the transcripts using ESTExplorer indicated that 290 (41%) sequences had homologues in *Caenorhabditis elegans*, 329 (42%) in parasitic nematodes, 202 (28%) in organisms other than nematodes, and 218 (31%) had no significant match to any sequence in the current databases. Of the *C. elegans *homologues, 90 were associated with 'non-wildtype' double-stranded RNA interference (RNAi) phenotypes, including embryonic lethality, maternal sterility, sterile progeny, larval arrest and slow growth. We could functionally classify 267 (38%) sequences using the Gene Ontologies (GO) and establish pathway associations for 230 (33%) sequences using the Kyoto Encyclopedia of Genes and Genomes (KEGG). Further examination of this EST dataset revealed a number of signalling molecules, proteases, protease inhibitors, enzymes, ion channels and immune-related genes. In addition, we identified 40 putative secreted proteins that could represent potential candidates for developing novel anthelmintics or vaccines. We further compared the automated EST sequence annotations, using ESTExplorer, with database search results for individual *T. vitrinus *ESTs. ESTExplorer reliably and rapidly annotated 301 ESTs, with pathway and GO information, eliminating 60 low quality hits from database searches.

**Conclusion:**

We evaluated the efficacy of ESTExplorer in analysing EST data, and demonstrate that computational tools can be used to accelerate the process of gene discovery in EST sequencing projects. The present study has elucidated sets of relatively conserved and potentially novel genes for biological investigation, and the annotated EST set provides further insight into the molecular biology of *T. vitrinus*, towards the identification of novel drug targets.

## Background

Many parasitic worms, including roundworms (nematodes), cause diseases in humans, animals and plants, which have substantial socio-economic impact throughout the world [1]. Investigating the molecular biology of such parasitic nematodes is not only of fundamental significance but could also lead to the discovery of novel methods for their control. In spite of the importance of parasitic nematodes [2], little is known and understood about them at the molecular level [3-5]. Clearly, molecular biological research, including whole genome and expressed sequence tag (EST) sequencing of key parasitic nematodes, provides a critically important foundation for a wide range of fundamental areas (including functional genomics, genetics, proteomics, systems biology, molecular biology, physiology, biochemistry, ecology, epidemiology, pathology and many more), underpinning many applied areas. Importantly, genomic technologies employed in an integrated way also have considerable potential for the identification of new drug targets linked to key biological pathways in parasitic nematodes of major socio-economic importance. This is of particular relevance, given the current problems with resistance against nematocidal drugs [6,7].

Genome sequencing of parasitic nematodes has focused predominantly on the use of a "global" EST approach [8,9]. Besides 'house-keeping' and structural genes isolated employing this approach, some genes that relate to drug target candidates have been identified in EST data sets. These include genes encoding antioxidant and de-toxifying enzymes, proteinases, proteinase inhibitors, cyclophilins, neurotransmitter receptors, transporters and nuclear hormone receptors [10]. Other studies [4,11] have used a much more "targeted" approach for exploring gender-enriched genes in *Trichostrongylus vitrinus *(an important trichostrongylid nematode of small ruminants) and *Oesophagostomum dentatum*, a nodule worm of pigs. For example, Nisbet and Gasser [4] investigated gender-enriched transcription in *T. vitrinus via *the construction of male- and female-enriched cDNA archives using suppressive-subtractive hybridization (SSH), sequencing of ESTs from these archives, comparison with genes of the free-living nematode, *Caenorhabditis elegans *and other organisms, and transcription profiling of a representative ESTs by array analysis. In the latter study, an emphasis was placed on a comparative analysis with data available for *C. elegans*, as it is currently the best-characterized nematode, (see WormBase [12]).

The whole genome sequencing approach is unlikely to be applied extensively to all organisms, irrespective of their commercial or scientific significance in human and animal health, agriculture and ecology. In contrast, expressed sequence tags (ESTs) offer a rapid and cost effective method to explore the transcriptome of an organism, leading to data representing short, unedited, single-pass sequence reads. ESTs are error prone and require several computational methods for pre-processing, clustering, assembly and annotation to yield biological information. As ESTs are usually generated in large numbers, it is crucial to be able to store, organize and annotate them using an automated analysis pipeline. A procedure is required to transfer data efficiently between programs without human intervention, based on carefully parameterized threshold criteria.

The analyses of EST data sets obtained [4,11] can take weeks or months if conducted manually, and the quality of analysis depends very much on the selection of software programs and packages employed. In order to address this limitation, we recently appraised current methods adopted for each step of EST analysis, compiled software tools considered most suited for EST pre-processing, clustering and assembly, database similarity searches and functional annotation, and proposed a "road map" to significantly accelerate the analyses of EST data sets [13]. After a detailed investigation of four current EST analysis platforms, ESTannotator [14], ESTAP [15], PartiGene [16], and EGassembler [17], a comprehensive workflow approach for gene and protein annotations was yet to be incorporated. Among the existing platforms, EGassembler terminates at the assembly level, providing contigs and singletons as output, whereas ESTSAP, ESTannotator and PartiGene provide predominantly an annotation at the nucleotide level. We have developed a semi-automated, user-definable computational workflow system, ESTExplorer [18], as a comprehensive workflow system for species-specific EST data assembly and annotation at both the DNA and protein levels.

In the present study, using a well-defined data set generated previously and analysed manually from the adult stage of a parasitic nematode, *T. vitrinus *[4], we evaluated the efficacy of ESTExplorer to assemble and correctly annotate ESTs. Compared with the previous study [4], which was limited to the annotation of ESTs *via *similarity searches (BLAST) for assigning putative function, we have added annotations to the EST data and identified important genes for further investigation. Firstly, we have included functional identification, in terms of mapping to protein domains and metabolic pathways. Secondly, we have categorised the *T. vitirnus *ESTs based on comparison with three databases (Wormpep [12], parasitic nematodes database and non-nematodes database (parasitic nematodes database and non-nematodes database are locally built databases) and used the Java tool SimiTri [19] to visualize the data comparison. Thirdly, we have related ESTs to molecules in *C. elegans *which can be silenced by double-stranded RNA interference (RNAi). For comparison purposes, we have repeated the earlier EST analysis [4] by running BLAST against the current databases, in order to minimize any database bias in the results, and compared these results with the automated EST sequence annotations for *T. vitrinus *data. We showed that ESTExplorer provides a comprehensive, but controlled functional annotation for EST datasets, which leads to a deeper understanding of the annotated molecules. Finally, we have identified 40 putative secreted proteins which represent potential candidates for developing novel anthelmintics or vaccines, using a procedure developed for the EST analysis for the bovine lungworm *Dictyocaulus viviparus *[20].

## Results and discussion

### Overall EST analysis

In total, 1776 sequences (866 female and 910 male) were obtained from 2112 clones selected from the gender-specific libraries [4]. The pre-processed male ESTs ranged from 101–585 bp in length (with a mean of 328 bp), and 103–695 bp (with a mean of 340 bp) for female ESTs. After clustering, the mean length of the contigs (or consensus sequences) increased to 412 (+/- 84) bp and 430 (+/-102) bp for the male-specific and female-specific datasets, respectively. The G+C contents of the coding sequences for male and female ESTs were 45.9% and 46.2%, respectively, which is consistent with other related ("clade V") nematodes [21], and higher than for *C. elegans *(37%) and *C. briggsae *(38%) [22].

We provided *T. vitrinus *male and female EST sequences separately as input to ESTExplorer for analyses, using all three phases. The CPU time taken for the processing of all of the programs (Phases I to III) in ESTExplorer is given in Table 1. All programs were run on a 16 CPU Linux cluster. It took 5158 sec (1 h 43 min) to process 910 male ESTs and 3861 seconds (1 h 07 min) to process 866 ESTs from female dataset. The time taken to assemble and annotate EST data was substantially reduced compared with previous manual analysis [4], for which individual ESTs were submitted for BLAST analysis, taking weeks to perform.

**Table 1:** Time taken for *Trichostrongylus vitrinus *EST data analysis carried out on 16 CPU Linux cluster (CPU time in seconds).

**Category**	**Number of ESTs**	**Sequential BLASTX alone (s)**	**ESTExplorer (s)**							
			
			**PHASE I**			**PHASE II**	**PHASE III**			
			
			**SeqClean**	**RepeatMasker**	**CAP3**	**BLASTX + BLAST2GO**	**ESTScan**	**InterProScan (12 programs)**	**KOBAS**	**Total time taken in seconds**
Male	910	981	8	52	48	1218	4	2461	1367	**5158**
Female	866	729	7	47	45	918	3	1867	974	**3861**

**Figure 1:**
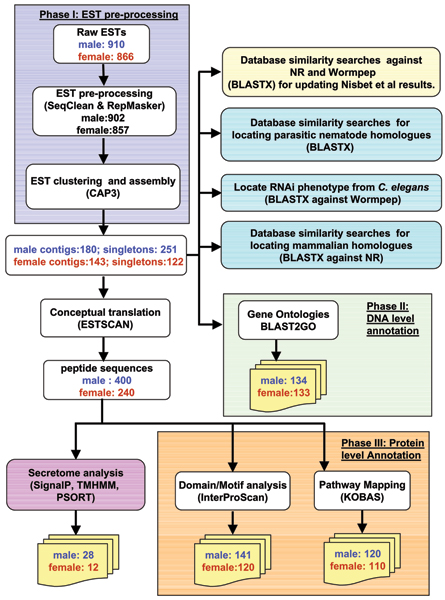
Bioinformatics analysis schema for *T. vitrinus *ESTs.

The cluster analysis of the 902 male and 857 female ESTs from *T. vitrinus *yielded 431 male and 265 female representative ESTs (rESTs; 180 male contigs and 251 singleton sequences; female 143 contigs and 122 singleton sequences; see Figure 1), of which 400 (92.8%) male and 240 (90.1%) female sequences had open reading frames (ORFs). Of a total of 640 peptides obtained by conceptual translation, 161 peptides could be mapped to either a protein domain or a motif. Figure 1 shows the detailed schema of all the programs used during the analysis, together with the mapping results for male and female EST datasets.

Male-specific protein kinases and protein phosphatases, major sperm proteins and enzymes involved in carbohydrate metabolism were abundant in the male EST dataset. Female-specific vitellogenins, heat-shock proteins and chaperonins were also highly represented. Genes involved in a number of cellular processes, such as ubiquitination and proteasome function, gene transcription, cell signaling, protein-protein interactions and chromatin assembly and function were also transcribed in a gender-specific manner. The gender-specific or gender-enriched transcription of many of the genes in the analyses is likely to reflect their roles in energy provision, gametogenesis, embryogenesis and reproduction, as discussed previously [4,11].

**Figure 2:**
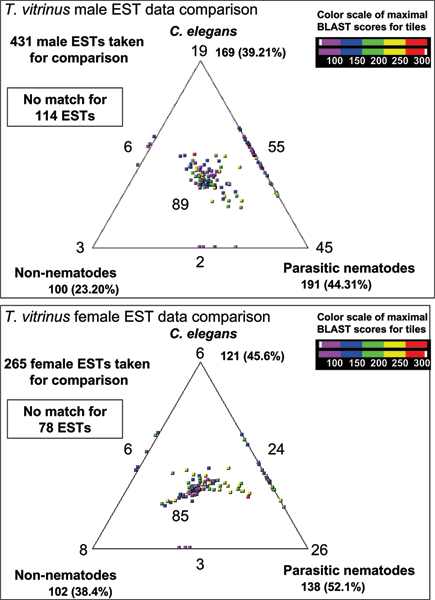
Comparison of male *Trichostrongylus vitrinus *ESTs with *Caenorhabditis elegans*, parasitic nematodes and non-nematodes protein sequence databases. Quantitative analysis of degree of similarity between *T. vitrinus *sequences homologous to those from *C. elegans*, other nematodes and non-nematode sequences, visualized by SimiTri. These comparisons demonstrate that the sampled transcriptome from male and female *T. vitrinus *has a closer relationship to molecules in parasitic nematodes and *C. elegans *than to non-nematodes.

Representative ESTs (rESTs) were also queried against three databases containing protein sequences from different organisms, in order to categorize the molecules from the *T. vitrinus*. Data were compared with protein sequences available for (i) *C. elegans *(from WORMPEP v.167 [12]), (ii) parasitic nematodes (available protein sequences and peptides from conceptually translated ESTs) and (iii) organisms other than nematodes (from NCBI non-redundant protein database) [23]. A three-way comparison of male and female *T. vitrinus *rESTs with homologues from *C. elegans*, WORMPEP and parasitic nematodes has been figuratively presented using SimiTri [19] (Figure 2). A total of 696 rESTs from male and female *T. vitrinus *were taken for this comparison. For the male, we found 169 (39.21%) homologues to *C. elegans*, 191 (44.31%) to those from other parasitic nematodes, including some strongylids, 100 (23.20%) homologues in organisms other than nematodes, and 114 (36.5%) with no significant similarity to any other organism (cut-off of <0.00001 [10^-5^]) for which sequence data are currently available (Figure 2).

From the comparative analysis of 265 female rESTs, we found 121 (45.6%) homologues to *C. elegans*, 138 (52.1%) to those from other parasitic nematodes, including some strongylids, 102 (38.4%) homologues in organisms other than nematodes and 78 (29.4%) with no significant similarity to any other organism for which sequence data are currently available. The SimiTri plot (Figures 2) shows, using the current archive, that the transcriptome subset from *T. vitrinus *has a closer relationship to that of other parasitic nematode and *C. elegans *than to that of non-nematodes.

The comparative analysis to identify homologues in *C. elegans *is important because *T. vitrinus *and this free-living nematode are both considered to belong to clade V of the Nematoda [24], and because *C. elegans *also represents the best characterized nematode in many respects, particularly in terms of its genome, genetics, biology, physiology, biochemistry, and the localization and function of molecules [12,25]. Specifically, the comparative analysis (at the amino acid level) of all rESTs with the *C. elegans *data revealed 169 (39.21%) sequences in male and 121 (45.6%) sequences in female to be key, well-characterized molecules associated with a range of important biological processes (male n = 80 and female n = 88), including development, regulation of biological processes, metabolic process, response to abiotic and biotic stimuli and reproduction. 'Non-wildtype' RNAi phenotypes in *C. elegans *homologues, such as Bmd (body morphology defect), Clr (clear), Emb (embryonic lethal), Gro (slow growth), Muv (multivulva), Mlt (molt defect), Slu (sluggish), Stp (sterile progeny), Sck (sick), Let (larval lethal), Lvl (larval lethal), Lva (larval arrest) and Unc (uncoordinated); (see WormBase [26] for details) were characterized for 30 and 60 molecules from male and female *T. vitrinus*, respectively (Additional File 1).

Of all 696 rESTs, the functions for 267 (62%) sequences could be predicted using descriptions from Gene Ontology (GO) [27], with 230 (33%) sequences mapping to key biological pathways (including signal transduction mechanisms, antigen processing and presentation, and/or the regulation of actin cytoskeleton, ribosomal proteins and translation factors). Overall, the functional classification revealed that approximately half of the rESTs had homologues in *C. elegans *and parasitic nematodes, nearly half of the sequences had homologues in other parasitic nematodes and one third of the rESTs did not have significant similarity to any of the sequences in current databases, including those with known functional domains, thus possibly representing novel genes.

### RNAi phenotypes in *C. elegans*

The use of genomic approaches, such as RNAi, transgenesis and microarray technologies, can considerably accelerate the characterisation of novel genes [9]. *C. elegans *(non-wild-type) RNAi phenotypes can provide an indication of the relevance and functions of orthologous genes in other nematodes, particularly in parasitic nematodes of clade V, for which the complexity of an obligate parasitic life cycle and the lack of an effective (long-term) laboratory culture system make high-throughput functional screening impractical [28].

We retrieved the *C. elegans *RNAi data representing *T. vitrinus *orthologues. Of 431 male and 265 female *T. vitrinus *rESTs, 30 (18.3%) male and 60 (49.5%) female sequences had homologues in *C. elegans *which have been silenced by RNAi (see Additional File 1). The RNAi phenotypes (as listed in Wormbase) included Adl (adult lethal), Age (ageing alteration), Bmd (body morphology defect), Dpy (dumpy), Egl (egg laying defect), Emb (embryonic lethal), Gro (slow growth), Let (larval lethal), Lvl (larval lethal), Lva (larval arrest) and Unc (uncoordinated).

In parasitic nematodes, successful RNAi has been reported in some plant parasites, such as *Heterodera glycines *and *Globodera pallida, *and in the animal parasites, *Nippostrongylus brasiliensis*, *Brugia malayi*, *Onchocerca volvulus*, *Ascaris suum *and *Trichostrongylus colubriformis *[29-34]. Geldhof *et al*. [35] have described that, under certain conditions, it is possible to silence some genes in *Haemonchus contortus *by RNAi. From the present study, we have predicted 90 rESTs from *T. vitrinus *to have homologues in *C. elegans *which can be silenced by RNAi. Possible targets for intervention are molecules with the phenotypes adult lethal, embryonic lethal, larval lethal and/or larval arrest. With drug resistance in strongylid parasites increasing, the development of an effective RNAi approach in parasitic nematodes could provide a powerful tool for the functional characterization of molecules involved in development, reproduction and survival, and could potentially lead to identification of novel therapeutic targets. Nonetheless, significant efforts are still required to develop a reliable and reproducible RNAi approach for a number of species [28,36].

### Gene Ontologies

GO has been used widely to predict gene function and classification [27]. It provides a dynamic vocabulary and hierarchy that unifies descriptions of biological, cellular and molecular functions across genomes. We used BLAST2GO [37], a sequence-based tool, to assign GO terms, extracting them for each BLAST hit obtained by mapping to extant annotation associations. We found that GO terms could be functionally assigned to 134 (31%) male and 133 50(%) female sequences of 696 (both male and female) rESTs. In summary, we found the following GO terms for male and female sequences: male, biological processes (n = 80), cellular components (n = 45) and molecular functions (n = 77) and female, biological processes (n = 88), cellular components (n = 62) and molecular functions (n = 83). A summary GO representation of the *T. vitrinus *rESTs is given in Additional File 2.

### Gene Ontologies for male *T. vitrinus* sequences

Amongst the most common GO categories in biological processes were developmental process (GO: 0032502), metabolic process (GO: 0008152), reproduction (GO: 0000003) and growth (GO: 0040007). Binding (GO: 0005488), catalytic activity (GO: 0003824) and structural molecule activity (GO: 0005198) were most common GO categories representing molecular function. The largest number of GO terms in cellular components was for cell part (GO: 0044464), membrane-bound organelle (GO: 0043227) and non-membrane-bound organelle (GO: 0043228). A complete listing of male GO mappings assigned to rESTs is given in Additional File 2.

### Gene Ontologies for female *T. vitrinus* sequences

We found similar GO categories in female sequences, including developmental process (GO: 0032502), metabolic process (GO: 0008152), reproduction (GO: 0000003), growth (GO: 0040007) and multicellular organismal process (GO: 0032501) for biological processes. The category, GO: 00032501, is not found in the male ESTs analysed in this study. Binding (GO: 0005488), catalytic activity (GO: 0003824) and structural molecule activity (GO: 0005198) were the most common GO categories representing molecular function. The largest number of GO terms in cellular components was for cell part (GO: 0044464), membrane-bound organelle (GO: 0043227) and non-membrane-bound organelle (GO: 0043228). A complete listing of female GO mappings assigned to rESTs is given in Additional File 2.

### Pathway analysis using KEGG assignments

Biochemical functionality was predicted by mapping male (431) and female (265) molecules to pathways, using KOBAS implemented within ESTExplorer [38], with an E-value cut-off of 1.0 e-5. After mapping sets of rESTs to pathways, we have then collected the enzymes (including EC numbers) within each pathway for possible biochemical assays. In the case of male molecules, a total of 120 (28%) sequences were mapped to 53 KEGG pathways, with 100 sequences representing metabolic enzymes characterized by unique EC numbers. A higher percentage of female molecules could be mapped. Out of 265 female molecules, a total of 110 (41%) sequences were mapped to 57 KEGG pathways, with 78 sequences representing metabolic enzymes characterized by unique EC numbers. KEGG biochemical pathway mapping data for male and female are listed in Additional File 3.

Amongst the rESTs mapped to KEGG pathways, molecules involved in glycolysis/gluconeogenesis, pyruvate metabolism and the proteasome had the highest representation amongst the male sequences, whereas members of ribosomal subunits were highly represented in female sequences as well as pyruvate metabolism and aminosugar metabolism. We identified seven putative proteins with potential roles in host-parasite interactions, such as molecules predicted to be involved in antigen processing and/or presentation, T-cell receptor signalling pathway and CD molecules. We found nine pathways considered functionally crucial for the organisms, such as signal transduction mechanisms, apoptosis and ubiquitin-mediated proteolysis.

### Secretome analysis

Important in the identification of potential novel drug or vaccine candidates in parasites is the prediction of molecules that are secreted or excreted in or around the host parasite interface [39-41]. Examples of such proteins are the aspartyl protease inhibitor API-1 [42], mi-msp-1 (similar to the venom allergen antigen AG5-like protein) [43] and the *Ancylostoma*-secreted protein (ASP) [44].

From the present data set (431 male and 265 female ESTs), we predicted 40 secreted proteins representing a non-redundant catalogue of *T. vitrinus *molecules (Table 2). Of these, 24 (60%) had homologues in nematodes, with 14 (35%) homologues in *C. elegans *and/or *C. briggsae *and 5 (12%) in various other parasitic nematodes, including the trichostrongylids *Haemonchus contortus *and *Trichostrongylus colubriformis *as well as the filarioid *Brugia malayi*. Of the 40 proteins inferred to be secreted, 16 (40%) were novel (sequences with no significant similarity to any sequence in current databases), making them intriguing candidates for further characterization, because they may relate specifically to parasitism. The secretome analysis revealed some very interesting molecules. For example, the protein predicted from TvmContig101 (from male) has homology to an astacin 13 in *C. elegans *[45]. The astacins are zinc metalloproteases present in prokaryotic and eukaryotic organisms and serve a variety of physiological functions, such digestion, hatching, peptide processing and morphogenesis [45]. TVmContig96 is predicted to code a protein which has homology to RPN-1, a non-ATPase subunit of the 19S regulatory particle (RP) of the 26S proteasome. RPN-1 is required for embryonic, larval and germline development and by homology, is predicted to function in unfolding and recognition of protein substrates and/or recycling of ubiquitin moieties during protein degradation [46].

**Table 2:** Identification and analysis of secreted proteins from *Trichostrongylus vitrinus *ESTs.

**Number**	**EST sequence ID**	**Seq Length (aa)**	**Start**	**Signal Peptide length (aa)**	**Description (top hit from non-redundant protein database)**	**E-value**	**% Identity (aa)**	**RNAi phenotype in *C. elegans***
1	TvmContig8	102	-	19	No significant hits	-	-	-
2	TVmContig11	148	-	24	No significant hits	-	-	-
3	TVmContig20	121	-	25	24 kDa excretory/secretory protein [*Haemonchus contortus*]	3.00E-028	63/109 (57%)	
4	TvmContig28	167	-	19	No significant hits	-	-	-
5	TVmContig35	120	M	17	30 kDa antigenic glycoprotein precursor [*Trichostrongylus colubriformis*]	4.00E-006	27/65 (41%)	No observed phenotype is found.
6	TVmContig39	208	-	18	Weakly similar to PhosphoDiEsterase family member (pde-2) [*Caenorhabditis elegans*]	4.00E-028	22/72 (30%)	No observed phenotype is found.
7	TvmContig68	131	-	26	Weakly similar to SaPosin-like Protein family member (spp-19) [*Caenorhabditis elegans*]	0.016	23/82 (28%)	No observed phenotype is found.
8	TVmContig75	239	-	26	Weakly similar to Na/Ca eXchangers family member (ncx-4) [*Caenorhabditis elegans*]	0.3	15/39 (38%)	No observed phenotype is found.
9	TVmContig96	120	-	15	Non-ATPase-like family member (rpn-1) [*Caenorhabditis elegans*]	2.00E-047	94/119 (78%)	EMB, LET embryonic_lethal, maternal_sterile, sick (Sck)
10	TvmContig101	156	M	18	Zinc metalloproteinase nas-13 precursor (Nematode astacin 13) [*Caenorhabditis elegans*]	2.00E-008	30/96 (31%)	No observed phenotype is found.
11	TVmContig107	85	-	27	No significant hits	-	-	-
12	TVmContig134	190	-	26	No significant hits	-	-	-
13	TvmContig135	116	-	19	Late Embryo Abundant (LEA) related family member (lea-1) [*Caenorhabditis elegans*]	4.00E-004	22/86 (25%)	No observed phenotype is found.
14	TVmContig150	137	-	24	No significant hits	-	-	-
15	TVmContig164	122	-	20	Weakly similar to Human xnp gene related protein 1 [*Caenorhabditis elegans*]	0.18	18/57 (31%)	None
16	TVmContig170	85	M	19	No significant hits	-	-	-
17	TVmContig171	131	M	15	No significant hits	-	-	-
18	TVmContig172	129	-	26	Weakly similar to abnormal NUClease family member (nuc-1) [*Caenorhabditis elegans*]	0.016	12/30 (40%)	life span abnormal (Age)
19	TvmContig178	146	-	17	Weakly similar to Maternal Effect Sterile family member (mes-3) [*Caenorhabditis elegans*]	0.047	13/26 (50%)	STP sterile_progeny
20	TVm01_A12	91	-	18	No significant hits	-	-	-
21	TVm02_D11	86	M	24	No significant hits	-	-	-
22	TVm03_G11	108	-	20	No significant hits	-	-	-
23	TVm04_F02	97	-	19	No significant hits	-	-	-
24	TVm06_F08	72	-	25	No significant hits	-	-	-
25	TVm07_A07	78	-	26	No significant hits	-	-	-
26	TVm07_G02	124	-	16	putative calcium-binding mitochondrial carrier protein [*Brugia malayi*]	1.00E-036	75/116 (64%)	GRO slow_growth |Unclassified
27	TVm08_D01	64	M	19	Hypothetical protein MGG_00752 [*Magnaporthe grisea *70-15]	8.00E-006	25/52 (48%)	None
28	TVm08_H01	120	-	19	Pyruvate kinase [*Caenorhabditis elegans*]	6.00E-025	42/87 (48%)	EMB, LET embryonic_lethal
29	TvfContig1	152	-	22	No significant hits	-	-	-
30	TvfContig3	143	-	23	C-type LECtin family member (clec-88) [*Caenorhabditis elegans*]	5.00E-021	55/132 (41%)	No observed phenotype is found.
31	TvfContig5	134	M	22	Hypothetical protein K06A9.1c [*Caenorhabditis elegans*]	0.004	28/98 (28%)	None
32	TvfContig78	161	-	20	MSH (MutS Homolog) family member (msh-2) [*Caenorhabditis elegans*]	2.00E-027	70/167 (41%)	spontaneous mutation rate increased
33	TvfContig84	104	M	18	No significant hits	-	-	-
34	TvfContig88	145	M	22	similar to TPRXL protein [*Pan troglodytes*]	0.002	31/122 (25%)	None
35	TvfContig122	97	-	24	No significant hits	-	-	-
36	TvfContig132	214	M	13	Vitellogenin-6 precursor [*Caenorhabditis elegans*]			GRO slow_growth |EMB, LET embryonic_lethal
37	TVf03_C08	132	-	20	No significant hits	-	-	-
38	TVf04_D01	162	-	16	putative serine-threonine kinase PAR-4 [*Caenorhabditis elegans*]	0.027	16/35 (45%)	No observed phenotype is found.
39	TVf06_A07	145	-	22	15 kDa excretory/secretory protein [*Haemonchus contortus*]	6.00E-004	39/118 (33%)	No observed phenotype is found.
40	TVf07_A05	146	-	23	No significant hits	-	-	-

### Comparison of individual EST analysis with ensemble annotation by ESTExplorer

Previously, Nisbet and Gasser [4] carried out an individual BLAST analysis of the 1776 gender-enriched ESTs from *T. vitrinus *and categorised the molecules into different functional classes. This conventional analysis took ~16 weeks to perform compared with an enhanced analysis of the same data set using ESTExplorer (with the exception of the secretome, SimiTri and RNAi phenotype analyses) which took less than 3 h. A total of 301 ESTs representing 31 functional categories were defined and compared. Male *T. vitrinus *was represented predominantly by major sperm protein-like, protein kinases/phosphatases, transcription factors, nucleic acid synthesis and other categories (Table 3), whereas female *T. vitrinus *was represented by vitellogenins, protein kinases/phosphatases and transcription factor and molecules involved in carbohydrate metabolism and modification and the ubiquitin-proteasome pathway (Table 3).

**Table 3:** Comparision of annotations for *T. vitrinus *ESTs: EST annotations performed by Nisbet and Gasser [4] based on BLAST similarity results in first three columns and annotations obtained from ESTExplorer are shown in next columns. Example catagories selected for male ESTs are major sperm protein-like, protein kinases and protein phosphatases, transcription factors and related and nucleic acid synthesis and function and for female ESTs, vitellogenins, carbohydrate metabolism and modification, ubiquitin-proteasome pathway, protein kinases/phosphatases and transcription factors and related proteins

**Manual annotation using BLAST**			**Annotations obtained automatically from ESTExplorer**				
**MALE: Protein kinases and protein phosphatases**							
**EST ID**	**E-value**	**BLAST results**	**BLAST results**	**E-value**	**Gene Ontologies**	**Metabolic Pathway Mapping**	**Domain/Motif data**

TVm02_C07	2.00E-37	PP1-gamma serine/threonine protein phosphatase	protein phosphatasecatalyticgamma isoform isoform 1	1.00E-36	chromatin modification, protein amino acid dephosphorylation, embryonic cleavage, cytokinesis, meiosis, oviposition, manganese ion binding, protein phosphatase type 1 activity, mitochondrial outer membrane, protein binding, mitosis, glycogen metabolic process, iron ion binding, nucleus	Long-term potentiation, Regulation of actin cytoskeleton, Focal adhesion, Insulin signaling pathway	Metallophosphoesterase, Serine/threonine-specific protein phosphatase and bis(5-nucleosyl)-tetraphosphatase
TVm02_F11	2.00E-45	Serine/threonine protein kinase of the casein kinase I subfamily	tau tubulin kinase	1.00E-44	protein kinase activity, ATP binding, protein amino acid phosphorylation, locomotory behavior, growth, larval development (sensu Nematoda)	Nicotinate and nicotinamide metabolism, Inositol phosphate metabolism, Benzoate degradation via CoA ligation	Protein kinase
TVm08_H06	5.00E-60	Member of the protein phosphatase protein family	serine threonine protein phosphatase pp1	1.00E-64	phosphoprotein phosphatase activity, protein amino acid dephosphorylation, iron ion binding, manganese ion binding	None	Metallophosphoesterase, Serine/threonine-specific protein phosphatase and bis(5-nucleosyl)-tetraphosphatase
TVm09_B11	8.00E-34	Protein phosphatase-1 (PP1)	phosphoprotein phosphatase 1	1.00E-33	growth, larval development (sensu Nematoda), embryonic development ending in birth or egg hatching, hydrolase activity	None	Metallophosphoesterase, Serine/threonine-specific protein phosphatase and bis(5-nucleosyl)-tetraphosphatase

**MALE: Transcription factors and related**							

**EST ID**	**E-value**	**BLAST results**	**BLAST results**	**E-value**	**Gene Ontologies**	**Metabolic Pathway Mapping**	**Domain/Motif data**

TVm01_F11	8.00E-10	Transcription elongation factor (testes specific homologue in mouse)	transcription elongation factor a1	1.00E-19	regulation of transcription, DNA-dependent, RNA elongation, translation elongation factor activity, zinc ion binding, RNA polymerase II transcription factor activity, nucleus, DNA binding, defense response, transcription regulator activity, transcription, regulation of transcription, transcription factor activity, transcription elongation factor complex, protein binding, transcriptional elongation regulator activity, erythrocyte differentiation, positive regulation of transcription, DNA-dependent, general RNA polymerase II transcription factor activity, metal ion binding, nucleoplasm	None	None
TVm09_B03	1.00E-09	Protein containing six kelch motifs and one BTB/POZ domain	klhl10 protein	1.00E-10	larval development (sensu Nematoda), growth, protein binding	None	Kelch repeat

**FEMALE: Vitellogenins**							

**EST ID**	**E-value**	**BLAST results**	**BLAST results**	**E-value**	**Gene Ontologies**	**Metabolic Pathway Mapping**	**Domain/Motif data**

TVf01_C08	4.00E-25	Vitellogenin 6 precursor	vitellogenin structural genes (yolk protein genes) family member (vit-6)	1.00E-24	embryonic development ending in birth or egg hatching;P:determination of adult life span, lipid transporter activity	None	Lipid transport protein, N-terminal
TVf01_C11	7.00E-14	Vitellogenin 5 precursor	vitellogenin structural genes (yolk protein genes) family member (vit-6)	1.00E-25	embryonic development ending in birth or egg hatching;P:determination of adult life span, lipid transporter activity	None	Lipid transport protein, N-terminal

**FEMALE: Carbohydrate metabolism and modification**							

**EST ID**	**E-value**	**BLAST results**	**BLAST results**	**E-value**	**Gene Ontologies**	**Metabolic Pathway Mapping**	**Domain/Motif data**

TVf02_G06	7.70E-15	glyceraldehyde 3 phosphate dehydrogenase gpd-3C	glyceraldehyde-3-phosphate dehydrogenase	1.00E-18	cell wall chitin biosynthetic process, glutamine-fructose-6-phosphate transaminase (isomerizing) activity	None	Glyceraldehyde 3-phosphate dehydrogenase, Lipocalin
TVf02_H12	6.80E-24	glucosamine-fructose-6-phosphate aminotransferase	glutamine-fructose-6-phosphate transaminase 2	1.00E-32	fructose 6-phosphate metabolic process, glutamine-fructose-6-phosphate transaminase (isomerizing) activity	Aminosugars metabolism, Glutamate metabolism	Glutamine amidotransferase, class-II
TVf03_B07	5.80E-28	Pyruvate dehydrogenase alpha subunit	pyruvate dehydrogenase e1 alpha subunit	1.00E-62	oxidoreductase activity, acting on the aldehyde or oxo group of donors, disulfide as acceptor, metabolic process, C:mitochondrion	Pyruvate metabolism, Glycolysis/Gluconeogenesis, Valine leucine and isoleucine biosynthesis, Butanoate metabolism	Dehydrogenase, E1 component
TVf03_E01	2.80E-35	Pyruvate dehydrogenase	dihydrolipoamide acetyltransferase	1.00E-53	oxidoreductase activity	Pyruvate metabolism, Glycolysis/Gluconeogenesis, Valine leucine and isoleucine biosynthesis, Butanoate metabolism	Transketolase, central region
TVf06_E11	3.80E-49	gei-7 isocitrate lyase	malate synthase a	1.00E-50	embryonic development, F:acyltransferase activity, tricarboxylic acid cycle, isocitrate lyase activity, determination of adult life span, glyoxylate cycle	Pyruvate metabolism, Glyoxylate and dicarboxylate metabolism	Malate synthase
TVf09_D01	3.20E-08	Ribulose-phosphate 3 epimerase	ribulose-phosphate 3-epimerase	1.00E-17	metabolic process, catalytic activity	Pentose phosphate pathway	Ribulose-phosphate 3-epimerase

**FEMALE: Ubiquitin-proteasome pathway**							

**EST ID**	**E-value**	**BLAST results**	**BLAST results**	**E-value**	**Gene Ontologies**	**Metabolic Pathway Mapping**	**Domain/Motif data**

TVf02_H07	3.00E-28	Ubiquitin-like	ubiquitin family member (ubq-1)	1.00E-27	ribosome, larval development (sensu Nematoda), structural constituent of ribosome, reproduction, growth translation, protein modification process	Ribosome	Ubiquitin
TVf06_D11	1.30E-12	pbs-2 Proteasome A-type and B-type	20s proteasome beta subunit pbb2	1.00E-22	threonine endopeptidase activity, ubiquitin-dependent protein catabolism, proteasome core complex (sensu Eukaryota)	Proteasome	20S proteasome, A and B subunits
TVf10_F06	5.20E-07	ubiquitin activating enzyme related (uba-1)	ubiquitin-activating enzyme e1	1.00E-12	ATP binding, ubiquitin-protein ligase activity, ubiquitin activating enzyme activity	Ubiquitin mediated proteolysis	Ubiquitin-activating enzyme repeat

**FEMALE: Protein kinases/phosphatases**							

**EST ID**	**E-value**	**BLAST results**	**BLAST results**	**E-value**	**Gene Ontologies**	**Metabolic Pathway Mapping**	**Domain/Motif data**

TVf06_F11	1.40E-49	lin-2 erythrocyte membrane like protein	calcium calmodulin-dependent serine protein kinase (maguk family)	1.00E-51	synapse, guanylate kinase activity, cell adhesion, positive regulation of vulval development (sensu Nematoda), actin cytoskeleton, protein-tyrosine kinase activity, synaptosome, nucleotide binding, basolateral plasma membrane, protein amino acid phosphorylation, cytosol, cell junction, calmodulin binding, protein serine/threonine kinase activity, positive regulation of transcription from RNA polymerase II promoter	Tight junction, Neurodegenerative Disorders	Pkinase_Tyr, Guanylate_kin

**FEMALE: Transcription factors and related**							

**EST ID**	**E-value**	**BLAST results**	**BLAST results**	**E-value**	**Gene Ontologies**	**Metabolic Pathway Mapping**	**Domain/Motif data**

TVf01_C02	3.80E-18	ama-1 RNA polymerase II	rna polymerase ii largest subunit	1.00E-30	DNA-directed RNA polymerase activity, DNA binding, transcription from RNA polymerase II promoter, DNA-directed RNA polymerase II, core complex	Purine metabolism, Pyrimidine metabolism, RNA polymerase	RNA polymerase Rpb1, domain 1
TVf03_A03	9.70E-25	btf-1 helicase	tbp associated factor	1.00E-63	nucleic acid binding, helicase activity, ATP binding	Purine metabolism	Helicase, C-terminal
TVf04_E07	2.00E-09	ceh-5 Homeobox domain	similarity to lim-homeobox protein lmx1_mesau	1.00E-15	metal ion binding, sequence-specific DNA binding, transcription factor activity, regulation of transcription, multicellular organismal development DNA-dependent, nucleus	None	None

We showed that ESTExplorer provides a comprehensive "functional" annotation for EST datasets which leads to an enhanced characterization and understanding of the annotated molecules. When a gene has multiple predicted functions, it is possible to list them in advanced annotation protocols, such as GO and pathway mapping, which provides a comprehensive prediction for the molecule to underpin any further molecular, biochemical and/or biological investigations. For instance, the EST TVm02_C07, which is homologous to a serine/threonine protein phosphatase, has revealed the following GO terms: "chromatin modification, protein amino acid dephosphorylation, embryonic cleavage, cytokinesis, meiosis, oviposition, manganese ion binding, protein phosphatase type 1 activity, protein binding, mitosis, glycogen metabolic process, iron ion binding, mitochondrial outer membrane, nucleus," and has been predicted to be involved in three pathways, "long-term potentiation, regulation of actin cytoskeleton, focal adhesion and insulin signaling pathway". Other findings indicated that this gene can be silenced in *C. elegans*, leading to progeny with Egl (egg laying deficit), Emb (embryonic lethal) and/or Sck (sick) phenotypes. Being able to achieve silencing in *C. elegans *showed that this gene is central to the development, reproduction and/or survival of this free-living nematode. This information provided a basis for the detailed molecular characterization and transcriptional analysis of the full-length gene (*Tv-stp-1*) encoding this serine/threonine protein phosphatase (*Tv*-STP-1) from *T. vitrinus*. The findings of this study [47] indicated that there is relative conservation in features and function of the serine/threonine protein phosphatase characterized among *T. vitrinus*, *O. dentatum *and *C. elegans*, likely to have significant implications for exploring molecular reproductive and developmental processes in strongylid nematodes of socio-economic importance.

Using ESTExplorer, we also showed that is possible to directly visualise the GO output according to the class of molecule. Blast2GO [37] (a part of Phase II) allows a data file to be generated which summarizes different classes of molecules with their representations (See Additional File 4). This facility is particularly useful when large EST data sets are being subjected to annotation. By using the online B2GO Java tool [48], statistical analyses (e.g., enrichment analysis using the Fisher's exact test) of particular classes of molecules can be conducted and hierarchical gene ontology graphs produced. Furthermore, EST Explorer applies several filtration steps for the removal of possible false positive predictions, by setting relatively high homology threshold values. We showed that 39 (male) and 21 (female) ESTs were not annotated by ESTExplorer in comparison with manual annotation. We examined the BLAST results for these ESTs and found that most of them indeed represented alignments with relatively low homology and incomplete sequence coverage. Thus, these ESTs did not meet the E-value threshold, set at 1.0E-05 as the default in ESTExplorer. For example, TVf02_D08 (3.40E-04) was annotated as a vitellogenin 3 precursor by individual BLAST search alone. Even when the E-value threshold (1.0E-05) was exceeded, the length of the alignment and its coverage were used to eliminate low homology matches. This issue was observed more frequently in categories, such as hypothetical, uncharacterised proteins and other proteins whose function has not yet been ascribed. A detailed comparison of all of the categories is given in Additional File 5. In addition, as users can modify the E-value thresholds and the overlap (percent identity) cut-offs during the assembly of ESTs in ESTExplorer, there is control over the way in which a particular EST dataset is annotated, which can range from "stringent" to "less stringent" parameters. This point is particularly important when investigating "lesser known" species, such as *T. vitrinus*, for which no genome sequence or functional data are available. Taken together, we demonstrate that ESTExplorer provides reliable, detailed and in-depth annotations, such as Gene Ontologies, mapped pathways and protein domain/motif annotations compared to BLAST-based annotations, with one such prediction (TVm02_C07) validated by experimentation.

## Conclusion and future directions

In this study, we critically evaluated a semi-automated EST analysis platform, ESTExplorer and compared the results obtained using this platform with those of a previous individual BLAST search approach. We have demonstrated that ESTExplorer is capable of rapidly analysing data, and also show the accuracy of the annotation using test datasets for the nematode *T. vitrinus *(Strongylida).

In the present study, among all test ESTs (n = 1776), we identified 192 novel sequences (27.5%), with high confidence, with no known homologue to any nematode or mammalian sequence currently available in public databases. These molecules are particularly interesting, as they may represent genes that relate to the parasitic mode of existence or to the species (*T. vitrinus*). However, such molecules are currently difficult to investigate, as their functions cannot be predicted using current bioinformatics approaches. Nonetheless, there is huge scope for studying such molecules in the future, using a combination of genomic and proteomic approaches. Insights into such molecules and/or their interplay with the ruminant host could provide opportunities for developing novel methods of parasite intervention. Opportunities will be enhanced when the complete genome of *T. vitrinus *and a detailed characterization of the transcriptome become available. Having available the whole genome sequence for *T. vitrinus*, will also underpin meaningful proteomic analyses of differentially expressed proteins. Importantly, the future application of an integrated bioinformatic-genomic-phenomic-proteomic ("systems biology") approach, focusing on molecular processes, will enhance our understanding of the molecular biology of moulting, invasion of and establishment in the host, hypobiosis (arrested development), and sexual differentiation, maturation and behaviour of *T. vitrinus*. Clearly, progress in such fundamental areas could lead to the development of new ways of controlling parasitic nematode, by blocking or disrupting key biological pathways in them.

Extending the present study, we are in the process of developing a user-definable workflow system specifically for the analysis of EST data from parasitic nematodes. We plan to integrate additional functionalities to the current version of ESTExplorer. For instance, the query sequence comparison with data available in three databases and generation of a SimiTri triangle to visualize homologous data. The BLAST searches against *C. elegans *protein sequences (Wormpep) and automatic retrieval of RNAi phenotypic data. With 454 Life Science GS20 sequencing technology [49,50], providing an unprecedented rate of genomic and EST data at substantially lower costs than incurred in conventional sequencers, ESTExplorer facilitates the timely analysis of high throughput data, providing "high confidence annotation" at the DNA and protein levels, enriched with gene ontology, protein domain and pathway information.

## Materials and methods

The EST data set representing molecules from *T. vitrinus *was obtained previously *via *the sequencing of gender-enriched cDNA from archives generated using suppressive-subtractive hybridisation (SSH) [4]. These EST data were initially analysed and annotated using the automated EST analysis platform ESTExplorer [38], available at . In brief, the analyses comprised three phases. In phase I, all ESTs were pre-processed (SeqClean, RepeatMasker), aligned/clustered using the Contig Assembly Program CAP3, employing a minimum sequence overlap length "cut-off" of 30 bases and an identity threshold of 95% (in Phase I) for the removal of flanking vector and adapter sequences, followed by assembly. Phase II of the ESTExplorer led to GO inference, at the DNA-level annotation, using BLAST2GO [37]. In Phase III, rESTs were then conceptually translated into peptides using ESTScan. The ESTScan program requires a matrix, known as the "smat" file, generated from available mRNA data for a specific organism, for conceptual translation. When the smat file for a specific organism is not available in ESTScan, the nearest well-studied organism using NCBI Taxonomy is selected as a reference and its smat file is used for conceptual translation. As *T. vitrinus *mRNA data are very scanty, we have selected *C. elegans *as the nearest reference organism for generating a smat file, based on available 25,481 (as on 15 February 2007) cDNA sequences from *C. elegans *was used for comparative analysis. *C. elegans *was selected because it belongs to the same clade (V) of the Nematoda [24,51] as *T. vitrinus*. We validated this approach by taking 56 untranslated sequences (contigs and singletons) and attempted to look for homologous proteins using BLASTX. We found that 36 entries did not return any hits from the BLASTX output (Additional file 6), with 12 sequences showing no matches to nematode sequences. Of the eight sequences with nematode matches, only two could be considered significant. Thus, the use of *C. elegans *smat is considered to be ~96% accurate (54/56 sequences). The peptides were mapped *via *InterProScan (domain/motifs) and to respective pathways in *C. elegans *using KOBAS (KEGG Orthology-Based Annotation System). Peptides were also compared, using BLASTP, with the non-redundant protein sequence database from National Centre for Biotechnology Information (NCBI), as part of the generic ESTExplorer pipeline for systematic EST analysis and annotation.

Protein databases for 'parasitic nematodes' and 'non-nematodes' were built in-house for similarity searches. The former group contains all available protein sequences for parasitic nematodes and ESTs from GenBank (17th May 2007), translated into peptide sequences whereas the latter database comprises amino acid sequences from the complete non-redundant protein database NR (17th May 2007) excluding any from nematodes. Additionally, homologues to peptides inferred from rESTs were identified *via *comparisons against WormBase using BLASTX. Each EST of *T. vitrinus *was assigned a 'statistically significant' gene homologue if the E-value from the BLAST output of the sequence alignment was <0.00001 (10^-5^). Comparison (at the amino acid sequence level) of *T. vitrinus *rESTs with *C. elegans*, parasitic nematode and non-nematode protein sequence databases using SimiTri [19]. SimiTri provides a two-dimensional display and an analysis of relative similarity relationships of the dataset of interest to three different databases.

Secreted proteins were predicted from the inferred peptides using a combination of three programs, in order to minimize the number of false positive predictions. Firstly, SignalP 3.0 [52] was used to predict the presence of secretory signal peptides and signal anchors for each predicted protein. A signal sequence was considered present if predicted both by the artificial neural network and the hidden Markov model prediction approaches (SignalPNN and SignalP-HMM, available as options within SignalP). In order to exclude the erroneous prediction of putative transmembrane (TM) sequences as signal sequences, TMHMM [53], a membrane topology prediction program, was then applied. We further validated the list of secreted proteins, using extracellular localization, using PSORT [54]. The performance of this semi-automated approach was evaluated and compared with the previous, manual analysis [4] in terms of effectiveness, efficiency, detail and time.

### Comparison of individual EST analysis with ensemble annotation by ESTExplorer

In 2003, Nisbet and Gasser [4] carried out their analysis on an EST-by-EST basis, using BLASTX against the non-redundant protein and WormPep databases. We have employed the same dataset using BLASTX [55] searches (30 April 2007), to update the original BLAST results with current databases. We then subjected the same EST dataset, separately for male and female data, to a systematic analysis using Phase I (pre-processing, assembly and consensus generation), Phase II (nucleotide level annotation) and Phase III (protein level annotation) in the ESTExplorer pipeline [19] (with all underlying databases updated to 30 April 2007). The individual EST analysis results, using BLASTX alone, were compared with the output obtained *via *the automated ESTExplorer pipeline.

## Competing interests

The authors declare that they have no competing interests.

## Authors' contributions

SHN, RBG and SR conceived and designed the research plan and participated in all aspects of data collection and analysis. SHN conducted the analysis and SHN, RBG, AJN and SR interpreted the data. All authors contributed towards writing of the manuscript. All authors read and approved the final version.

## Supplementary Material

Additional file 1 Comparison of 696 rESTs from *Trichostrongylus vitrinus *with *Caenorhabditis elegans *proteome (Wormpep v 167). The table also provides corresponding RNAi phenotypic information. Click here for file

Additional file 2 Gene Ontology mappings for *Trichostrongylus vitrinus *rESTs. Click here for file

Additional file 3 Metabolic pathways in *Trichostrongylus vitrinus *mapped by Kyoto Encyclopedia of Genes and Genomes (KEGG). Click here for file

Additional file 4 An example output for functional classification of ESTs generated using the Blast2GO component of ESTEXplorer's Phase II – Nucleotide-level Analysis. Click here for file

Additional file 5 Comparison of individual EST analysis *via *BLAST searches and ensemble annotation by ESTExplorer. Click here for file

Additional file 6 Validation of *Caenorhabditis elegans *smat file used for ESTScan by BLASTX results for untranslated EST sequences. Click here for file
