# Supplementary material for: In silico analysis of expressed sequence tags from Trichostrongylus vitrinus (Nematoda): comparison of the automated ESTExplorer workflow platform with conventional database searches
Source: BMC Bioinformatics. 2008 Feb 13;9(Suppl 1):S10. doi: 10.1186/1471-2105-9-S1-S10 (PMC2259411; doi:10.1186/1471-2105-9-S1-S10)
Supplement: Additional file 1 — Comparison of 696 rESTs from Trichostrongylus vitrinus with Caenorhabditis elegans proteome (Wormpep v 167). The table also provides corresponding RNAi phenotypic information. [file 1471-2105-9-S1-S10-S1.doc]

**Additional File 1:** In *silico* analysis of expressed sequence tags (EST) from *Trichostrongylus vitrinus* (Nematoda): comparison of the automated ESTExplorer workflow platform with database searches.

Shivashankar H. Nagaraj, Robin B. Gasser, Alasdair J. Nisbet and Shoba Ranganathan

**Table S1: Comparison of 696 rESTs from *Trichostrongylus vitrinus* with *C. elegans* proteome (Wormpep v 167). The table also provides corresponding RNAi phenotypic information.**

| **Number** | **Sequence ID** | **Number of ESTs** | **E-value** | **% identity (aa)** | **Description of *C. elegans* homologue** | **WB Gene ID** | **RNAi phenotype in *C. elegans*** |
| --- | --- | --- | --- | --- | --- | --- | --- |
| 1 | TvmContig24 | 3 | 6.00E-041 | 67% | status: Confirmed TR:Q6BET1 protein_id:CAH04733.1 | WBGene00008780 | EMB,LET embryonic_lethal |
| 2 | TvmContig32 | 19 | 2.00E-071 | 56% | status: Confirmed TR:O02063 protein_id:AAB52899.1 | WBGene00020661 | LVA larval_arrest |PVL protruding_vulva |STP sterile_progeny |
| 3 | TvmContig34 | 2 | 2.00E-028 | 75% | locus:rpn-3 Diphenol oxidase A2 status:Confirmed SW:Q04908 protein_id:AAA27966.1 | WBGene00004460 | EMB,LET embryonic_lethal |
| 4 | TvmContig37 | 6 | 3.00E-017 | 53% | locus:gld-1 female germline-specific tumor suppressor status:Confirmed SW:Q17339 protein_id:CAB03417.1 | WBGene00001595 | EMB,LET embryonic_lethal |
| 5 | TvmContig38 | 7 | 1.00E-010 | 44% | status:Confirmed TR:Q6BET1 protein_id:CAH04733.1 | WBGene00008780 | EMB,LET embryonic_lethal |
| 6 | TvmContig42 | 3 | 6.00E-034 | 51% | locus:rpn-6 status:Confirmed TR:Q5DX45 protein_id:AAW88394.1 | WBGene00004462 | EMB,LET embryonic_lethal |
| 7 | TvmContig43 | 3 | 1.00E-055 | 67% | locus:gsp-2 status:Confirmed SW:P48727 protein_id:AAK18957.1 | WBGene00001748 | EMB,LET embryonic_lethal |
| 8 | TvmContig63 | 3 | 8.00E-025 | 34% | status:Partially_confirmed TR:Q18603 protein_id:CAA97779.1 | WBGene00008079 | GRO slow_growth |
| 9 | TvmContig64 | 2 | 2.00E-006 | 44% | chromo domain of heterochromatin protein status:Confirmed TR:Q19972 protein_id:AAA83357.1 | WBGene00017990 | EMB,LET embryonic_lethal |
| 10 | TvmContig103 | 3 | 1.00E-042 | 82% | locus:gpi-1 status:Confirmed TR:Q7K707 protein_id:CAE54920.1 | WBGene00013597 | EMB,LET embryonic_lethal |
| 11 | TvmContig117 | 12 | 2.00E-031 | 44% | status:Confirmed TR:Q6BET1 protein_id:CAH04733.1 | WBGene00008780 | EMB,LET embryonic_lethal |
| 12 | TvmContig133 | 3 | 4.00E-050 | 85% | cytochrome C status:Confirmed SW:P19974 protein_id:AAB92035.1 | WBGene00017121 | GRO slow_growth |EMB,LET embryonic_lethal |
| 13 | TvmContig140 | 2 | 2.00E-048 | 95% | locus:rpt-1 26S protease regulatory subunit 7 status:Confirmed SW:Q18787 protein_id:CAB01414.1 | WBGene00004501 | EMB,LET embryonic_lethal |maternal_sterile |
| 14 | TVm01_E04 | 1 | 4.00E-013 | 53% | aldehyde reductase status:Partially_confirmed TR:Q17568 protein_id:AAB37733.1 | WBGene00015307 | EMB,LET embryonic_lethal |
| 15 | TVm01_H02 | 1 | 8.00E-008 | 53% | status:Confirmed TR:Q86DA0 protein_id:CAD90182.1 | WBGene00012803 | LVA larval_arrest |
| 16 | TVm03_C07 | 1 | 1.00E-020 | 50% | locus:star-2 status:Partially_confirmed TR:Q65CM6 protein_id:AAU20839.1 | WBGene00006423 | GRO slow_growth |LET,LVL larval_lethal |PRL paralyzed |SLU sluggish |
| 17 | TVm04_B04 | 1 | 1.00E-064 | 81% | status:Confirmed TR:Q2V4S6 protein_id:ABB88235.1 | WBGene00016415 | GRO slow_growth |locomotion_abnormal |
| 18 | TVm04_C06 | 1 | 2.00E-007 | 33% | locus:ajm-1 status:Partially_confirmed | WBGene00000100 | GRO slow_growth |EMB,LET embryonic_lethal |locomotion_abnormal |
| 19 | TVm04_C09 | 1 | 1.00E-007 | 82% | status:Confirmed TR:O02063 protein_id:AAB52899.1 | WBGene00020661 | LVA larval_arrest |PVL protruding_vulva |STP sterile_progeny |
| 20 | TVm04_E02 | 1 | 1.00E-062 | 98% | locus:vha-13 ATP synthase alpha and beta subunits \; ATP synthase ab C terminal status:Confirmed SW:Q9XW92 protein_id:CAA22076.1 | WBGene00013025 | GRO slow_growth |EMB,LET embryonic_lethal |maternal_sterile |
| 21 | TVm06_F02 | 1 | 2.00E-036 | 78% | locus:gpd-2 status:Confirmed SW:P17329 protein_id:AAB53874.1 | WBGene00001684 | EMB,LET embryonic_lethal |small |gonad_development_abnormal |
| 22 | TVm06_G03 | 1 | 4.00E-013 | 50% | status:Confirmed TR:Q19007 protein_id:AAA81494.1 | WBGene00017075 | GRO slow_growth |EMB,LET embryonic_lethal |
| 23 | TVm07_C10 | 1 | 1.00E-034 | 95% | locus:tbb-1 tubulin beta-chain status:Confirmed TR:O17921 protein_id:CAB07246.1 | WBGene00006536 | EMB,LET embryonic_lethal |
| 24 | TVm07_G02 | 1 | 5.00E-047 | 76% | mitochondrial carrier protein status:Partially_confirmed TR:Q93717 protein_id:CAB02107.1 | WBGene00009666 | GRO slow_growth |Unclassified |
| 25 | TVm08_A12 | 1 | 9.00E-007 | 52% | status:Confirmed TR:O02063 protein_id:AAB52899.1 | WBGene00020661 | LVA larval_arrest |PVL protruding_vulva |STP sterile_progeny |
| 26 | TVm08_H01 | 1 | 2.00E-020 | 48% | Pyruvate kinase status:Confirmed TR:Q3S1I8 protein_id:CAJ30227.1 | WBGene00009126 | EMB,LET embryonic_lethal |
| 27 | TVm09_G05 | 1 | 1.00E-008 | 39% | locus:star-2 status:Partially_confirmed TR:Q65CM6 protein_id:AAU20839.1 | WBGene00006423 | GRO slow_growth |LET,LVL larval_lethal |PRL paralyzed |SLU sluggish |
| 28 | TVm10_B02 | 1 | 2.00E-011 | 40% | casein kinase status:Partially_confirmed TR:Q22203 protein_id:AAA81115.1 | WBGene00020223 | GRO slow_growth |
| 29 | TVm11_D01 | 1 | 1.00E-036 | 74% | status:Confirmed TR:Q20121 protein_id:AAC48292.3 | WBGene00018152 | STP sterile_progeny |
| 30 | TVm11_G01 | 1 | 5.00E-020 | 44% | status:Partially_confirmed TR:Q9GUP2protein_id:AAG23375.2 | WBGene00022069 | EMB,LET embryonic_lethal |
|  | **Female sequences** |  |  |  |  |  |  |
| 1 | TvfemContig4 | 3 | 2.00E-019 | 42% | status:Confirmed TR:Q7JMK4 protein_id:CAE48493.1 | WBGene00007332 | EMB,LET embryonic_lethal |HIM high_incidence_male_progeny |
| 2 | TvfemContig17 | 2 | 1.00E-040 | 60% | eukaryotic peptide chain release factor subunit 1 status:Confirmed SW:O16520 protein_id:AAB66012.1 | WBGene00020269 | EMB,LET embryonic_lethal |maternal_sterile |
| 3 | TvfemContig25 | 3 | 2.00E-025 | 90% | locus:tag-61 ADP\/ATP carrier protein status:Confirmed TR:O45865 protein_id:CAB04874.1 | WBGene00006439 | EMB,LET embryonic_lethal |
| 4 | TvfemContig27 | 3 | 1.00E-008 | 25% | locus:gei-11 Myb DNA-binding proteins (2 domains) status:Partially_confirmed TR:O62208 protein_id:CAB04248.1 | WBGene00001568 | GRO slow_growth |EMB,LET embryonic_lethal |
| 5 | TvfemContig28 | 7 | 2.00E-052 | 56% | locus:try-1 Plasminogen status:Confirmed TR:Q23528 protein_id:AAA68746.2 | WBGene00006619 | EMB,LET embryonic_lethal |
| 6 | TvfemContig34 | 5 | 4.00E-015 | 33% | locus:vit-3 status:Partially_confirmedSW:Q9N4J2 protein_id:AAK09075.1 | WBGene00006927 | GRO slow_growth |EMB,LET embryonic_lethal |
| 7 | TvfemContig50 | 16 | 3.00E-033 | 38% | locus:rme-2 LDL-like receptor status:Confirmed TR:O44191 protein_id:AAB88554.3 | WBGene00004374 | EMB,LET embryonic_lethal |maternal_sterile |
| 8 | TvfemContig51 | 4 | 4.00E-009 | 33% | locus:cct-4 T-complex protein status:Confirmed SW:P47208 protein_id:CAA88861.1 | WBGene00000379 | EMB,LET embryonic_lethal |maternal_sterile |
| 9 | TvfemContig53 | 5 | 2.00E-024 | 38% | locus:vit-4 status:Partially_confirmedSW:P18947 protein_id:AAK09074.1 | WBGene00006928 | GRO slow_growth |EMB,LET embryonic_lethal |
| 10 | TvfemContig56 | 3 | 9.00E-015 | 40% | locus:gpd-2 status:Confirmed SW:P17329 protein_id:AAB53874.1 | WBGene00001684 | EMB,LET embryonic_lethal |small |gonad_development_abnormal |
| 11 | TvfemContig65 | 2 | 2.00E-020 | 88% | locus:unc-32 TJ6\/proton pump status:Confirmed SW:P30628 protein_id:CAD30453.1 | WBGene00006768 | EMB,LET embryonic_lethal |
| 12 | TvfemContig67 | 11 | 1.00E-068 | 78% | locus:tag-61 ADP\/ATP carrier protein status:Confirmed TR:O45865 protein_id:CAB04874.1 | WBGene00006439 | EMB,LET embryonic_lethal |
| 13 | TvfemContig69 | 2 | 1.00E-033 | 50% | glucosamine-fructose-6-phosphate aminotransferase status:Confirmed TR:Q95QM8 protein_id:CAC42276.1 | WBGene00008546 | EMB,OCS one_cell_arrest_early_emb |EMB,LET embryonic_lethal |maternal_sterile |
| 14 | TvfemContig71 | 9 | 1.00E-045 | 37% | locus:vit-4 status:Partially_confirmedSW:P18947 protein_id:AAK09074.1 | WBGene00006928 | GRO slow_growth |EMB,LET embryonic_lethal |
| 15 | TvfemContig72 | 2 | 3.00E-029 | 43% | status:Confirmed TR:Q8I111 protein_id:CAD59156.1 | WBGene00011510 | GRO slow_growth |EMB,LET embryonic_lethal |CLR clear |
| 16 | TvfemContig76 | 2 | 2.00E-006 | 34% | locus:mnat-1 status:Confirmed TR:O17245 protein_id:AAB71017.3 | WBGene00018769 | EMB,LET embryonic_lethal |maternal_sterile |
| 17 | TvfemContig77 | 2 | 2.00E-071 | 77% | phosphoglucomutase status:Confirmed TR:Q21742 protein_id:AAA83163.1 | WBGene00019890 | EMB,LET embryonic_lethal |
| 18 | TvfemContig79 | 2 | 1.00E-018 | 35% | status:Confirmed TR:O44145 protein_id:AAB88324.2 | WBGene00016636 | EMB,LET embryonic_lethal |
| 19 | TvfemContig85 | 2 | 5.00E-053 | 58% | pyruvate dehydrogenase status:Confirmed SW:O44451 protein_id:AAB92024.2 | WBGene00015413 | GRO slow_growth |EMB,LET embryonic_lethal |
| 20 | TvfemContig91 | 2 | 2.00E-010 | 46% | locus:cct-1 T-complex protein status:Confirmed SW:P41988 protein_id:CAA91308.1 | WBGene00000377 | EMB,LET embryonic_lethal |maternal_sterile |
| 21 | TvfemContig100 | 2 | 8.00E-016 | 37% | locus:enol-1 status:Confirmed | WBGene00011884 | GRO slow_growth |EMB,LET embryonic_lethal |small |CLR clear |
| 22 | TvfemContig102 | 2 | 2.00E-013 | 94% | endoplasmin precursor (GRP94) status:Confirmed TR:Q22235 protein_id:CAA92973.1 | WBGene00011480 | GRO slow_growth |
| 23 | TvfemContig103 | 5 | 2.00E-026 | 48% | locus:rpl-32 60S ribosomal protein L32 status:Confirmed TR:Q22716 protein_id:CAA92757.1 | WBGene00004446 | LVA larval_arrest |
| 24 | TvfemContig104 | 3 | 1.00E-013 | 52% | locus:pbs-2 Proteasome A-type and B-type status:Confirmed TR:O62102 protein_id:CAB16855.1 | WBGene00003948 | EMB,LET embryonic_lethal |locomotion_abnormal |
| 25 | TvfemContig107 | 3 | 3.00E-009 | 40% | locus:pbs-2 Proteasome A-type and B-type status:Confirmed TR:O62102 protein_id:CAB16855.1 | WBGene00003948 | EMB,LET embryonic_lethal |locomotion_abnormal |
| 26 | TvfemContig112 | 4 | 2.00E-022 | 48% | locus:vit-3 status:Partially_confirmedSW:Q9N4J2 protein_id:AAK09075.1 | WBGene00006927 | GRO slow_growth |EMB,LET embryonic_lethal |
| 27 | TvfemContig120 | 2 | 8.00E-040 | 65% | locus:krs-1 status:Confirmed TR:Q2XN16 protein_id:AAX88810.1 | WBGene00002238 | GRO slow_growth |EMB,LET embryonic_lethal |small |BMD organism_morphology_abnormal |STP sterile_progeny |
| 28 | TvfemContig125 | 3 | 8.00E-034 | 100% | locus:ubq-2 UBQ-2 ubiquitin\; 60S Ribosomal protein L40 status:Confirmed | WBGene00006728 | EMB,LET embryonic_lethal |
| 29 | TvfemContig128 | 2 | 4.00E-007 | 35% | locus:his-12 histone H2A status:Predicted SW:P09588 protein_id:CAB05836.1 | WBGene00001886 | EMB,LET embryonic_lethal |EMB nuclear_morphology_alteration_early_emb |
| 30 | TvfemContig129 | 2 | 1.00E-024 | 38% | locus:rpl-19 60S ribosomal protein L19 status:Confirmed SW:O02639 protein_id:AAB53979.1 | WBGene00004431 | EMB,LET embryonic_lethal |EMB embryonic_terminal_arrest_variable_emb |LVA larval_arrest |maternal_sterile |
| 31 | TvfemContig130 | 2 | 2.00E-022 | 50% | locus:apn-1 AP endonuclease (family 2) status:Confirmed SW:Q10002 protein_id:CAA87789.2 | WBGene00000151 | EMB,LET embryonic_lethal |
| 32 | TvfemContig132 | 10 | 2.00E-034 | 37% | locus:vit-5 status:Partially_confirmed SW:P06125 protein_id:AAA83587.1 | WBGene00006929 | GRO slow_growth |EMB,LET embryonic_lethal |
| 33 | TvfemContig137 | 2 | 6.00E-019 | 35% | locus:lin-53 chromatin assembly factor 1 P55 subunit like status:Confirmed SW:P90916 protein_id:CAA19477.1 | WBGene00003036 | EMB,LET embryonic_lethal |LET,LVL larval_lethal |PVL protruding_vulva |
| 34 | TvfemContig138 | 2 | 2.00E-007 | 41% | locus:mcm-4 status:Confirmed TR:Q95XQ8 protein_id:AAK39605.1 | WBGene00003156 | EMB,LET embryonic_lethal |EMB nuclear_morphology_alteration_early_emb |
| 35 | TvfemContig140 | 3 | 1.00E-043 | 53% | locus:rnr-1 Ribonucleoside-disphosphate reductase large chain status:Confirmed SW:Q03604 protein_id:CAA79574.1 | WBGene00004391 | EMB,LET embryonic_lethal |
| 36 | TVf01_C02 | 1 | 2.00E-020 | 67% | locus:ama-1 RNA polymerase II status:Partially_confirmed SW:P16356 protein_id:AAA96158.2 | WBGene00000123 | EMB,LET embryonic_lethal |maternal_sterile |
| 37 | TVf01_D12 | 1 | 3.00E-050 | 88% | locus:pdi-2 status:Confirmed TR:Q8IG53 protein_id:AAN39682.1 | WBGene00003963 | EMB,LET embryonic_lethal |locomotion_abnormal |SLU sluggish |CLR clear |
| 38 | TVf02_D04 | 1 | 9.00E-013 | 41% | endoplasmin precursor (GRP94) status:Confirmed TR:Q22235 protein_id:CAA92973.1 | WBGene00011480 | GRO slow_growth |
| 39 | TVf02_E01 | 1 | 2.00E-010 | 48% | nucleoside diphosphate kinase status:Confirmed TR:Q93576 protein_id:CAB02101.1 | WBGene00009119 | EMB,LET embryonic_lethal |STP sterile_progeny |
| 40 | TVf02_E09 | 1 | 3.00E-034 | 48% | locus:air-2 protein kinase status:Confirmed TR:O01427 protein_id:AAB52459.2 | WBGene00000099 | EMB,LET embryonic_lethal |
| 41 | TVf03_F08 | 1 | 2.00E-048 | 77% | locus:sec-5 status:Partially_confirmed SW:Q22706 protein_id:CAA92702.1 | WBGene00004752 | EMB,LET embryonic_lethal |EMB integrity_of_membranous_organelles_abnormal_early__emb |
| 42 | TVf04_G02 | 1 | 6.00E-011 | 30% | glucosamine-fructose-6-phosphate aminotransferase status:Confirmed TR:Q19130 protein_id:CAA91315.1 | WBGene00008546 | EMB,OCS one_cell_arrest_early_emb |EMB,LET embryonic_lethal |maternal_sterile |CYK cytokinesis_fails_early_emb |EMB,MUL multiple_nuclei_early_emb |EMB polar_body_reabsorbed_one_two_early_emb |EMB one_cell_shape_abnormal_early_emb |EMB embryo_osmotic_press |
| 43 | TVf05_B04 | 1 | 5.00E-019 | 47% | Yeast D96591.6 protein like status:Confirmed TR:Q93638 protein_id:CAB03017.2 | WBGene00009266 | SCK sick |STP sterile_progeny |
| 44 | TVf05_G09 | 1 | 4.00E-035 | 50% | aminotransferase status:Confirmed TR:O61741 protein_id:AAC16992.2 | WBGene00015021 | EMB,LET embryonic_lethal |LVA larval_arrest |Unclassified |
| 45 | TVf06_A05 | 1 | 2.00E-017 | 33% | locus:mcm-5 CDC46 status:Confirmed SW:Q21902 protein_id:CAA90765.1 | WBGene00003157 | EMB,LET embryonic_lethal |
| 46 | TVf06_C03 | 1 | 6.00E-012 | 58% | locus:vit-4 status:Partially_confirmedSW:P18947 protein_id:AAK09074.1 | WBGene00006928 | GRO slow_growth |EMB,LET embryonic_lethal |
| 47 | TVf06_G10 | 1 | 3.00E-011 | 40% | locus:rps-0 40S ribosomal protein status:Confirmed SW:P46769 protein_id:CAA86061.1 | WBGene00004469 | EMB,LET embryonic_lethal |maternal_sterile |
| 48 | TVf07_A07 | 1 | 2.00E-011 | 48% | locus:vit-4 status:Partially_confirmedSW:P18947 protein_id:AAK09074.1 | WBGene00006928 | GRO slow_growth |EMB,LET embryonic_lethal |
| 49 | TVf08_H01 | 1 | 8.00E-046 | 81% | locus:ben-1 tubulin status:Partially_confirmed TR:Q18817 protein_id:CAB00853.4 | WBGene00000248 | EMB,LET embryonic_lethal |
| 50 | TVf09_H10 | 1 | 5.00E-028 | 57% | Rat insulin-like growth factorbinding protien complex acid labile chain likestatus:Partially_confirmed TR:P90920protein_id:CAB03182.1 | WBGene00010621 | EMB,LET embryonic_lethal |
| 51 | TVf10_B04 | 1 | 4.00E-006 | 42% | status:Confirmed TR:O17072 protein_id:AAK68346.1 | WBGene00017742 | LVA larval_arrest |PVL protruding_vulva |STP sterile_progeny |
| 52 | TVf10_B05 | 1 | 4.00E-029 | 50% | locus:cct-1 T-complex protein status:Confirmed SW:P41988 protein_id:CAA91308.1 | WBGene00000377 | EMB,LET embryonic_lethal |maternal_sterile |
| 53 | TVf10_B07 | 1 | 2.00E-033 | 93% | locus:lev-11 status:Confirmed | WBGene00002978 | LON long |SCK sick |LET,LVL larval_lethal |BMD organism_morphology_abnormal |maternal_sterile |
| 54 | TVf10_C05 | 1 | 6.00E-054 | 91% | Pyruvate kinase status:Confirmed TR:Q3S1I8 protein_id:CAJ30227.1 | WBGene00009126 | EMB,LET embryonic_lethal |
| 55 | TVf10_C07 | 1 | 1.00E-048 | 47% | status:Confirmed TR:Q7Z133 protein_id:AAP68939.1 | WBGene00018863 | GRO slow_growth |
| 56 | TVf10_C10 | 1 | 1.00E-017 | 64% | locus:rpl-43 status:Confirmed SW:Q9U2A8 protein_id:CAB54440.1 | WBGene00004456 | GRO slow_growth |EMB,LET embryonic_lethal |PCH patchy_coloration |
| 57 | TVf10_D12 | 1 | 1.00E-015 | 72% | locus:mec-7 beta tubulin status:Partially_confirmed SW:P12456 protein_id:AAB09092.1 | WBGene00003171 | EMB,LET embryonic_lethal |
| 58 | TVf10_E10 | 1 | 9.00E-018 | 66% | centromere\/microtubule binding protein status:Confirmed SW:O17919 protein_id:CAB07244.1 | WBGene00010478 | LVA larval_arrest |
| 59 | TVf10_F06 | 1 | 8.00E-008 | 53% | locus:uba-1 status:Confirmed TR:Q3S1J5 protein_id:CAJ30225.1 | WBGene00006699 | SCK sick |EMB,LET embryonic_lethal |maternal_sterile |
| 60 | TVf11_B06 | 1 | 7.00E-012 | 68% | locus:his-72 Core histone H2A\/H2B\/H3\/H4 status:Confirmed TR:Q9U281 protein_id:CAB11546.1 | WBGene00001946 | EMB,LET embryonic_lethal |
