# Supplementary material for: In silico analysis of expressed sequence tags from Trichostrongylus vitrinus (Nematoda): comparison of the automated ESTExplorer workflow platform with conventional database searches
Source: BMC Bioinformatics. 2008 Feb 13;9(Suppl 1):S10. doi: 10.1186/1471-2105-9-S1-S10 (PMC2259411; doi:10.1186/1471-2105-9-S1-S10)
Supplement: Additional file 2 — Gene Ontology mappings for Trichostrongylus vitrinus rESTs. [file 1471-2105-9-S1-S10-S2.doc]

**Additional File 2:** In *silico* analysis of expressed sequence tags (EST) from *Trichostrongylus vitrinus* (Nematoda): comparison of the automated ESTExplorer workflow platform with database searches.

Shivashankar H. Nagaraj, Robin B. Gasser, Alasdair J. Nisbet and Shoba Ranganathan

**Table S2: Gene Ontology mappings (using GO slim terms) for *Trichostrongylus vitrinus* clusters. Note that individual GO categories can have multiple mappings.**

**Male dataset**

a. Biological process

| **GO Graph Level** | **GO Term** | **Representation** | **% Representation of total** |
| --- | --- | --- | --- |
| **1** | **Biological process** | **80** | **2.84** |
| 2 | cellular process | 56 | 1.99 |
| 2 | developmental process | 33 | 1.17 |
| 2 | growth | 19 | 0.67 |
| 2 | multicellular organismal process | 31 | 1.10 |
| 2 | reproduction | 20 | 0.71 |
| 2 | metabolic process | 42 | 1.49 |
| 2 | response to stimulus | 17 | 0.60 |
| 2 | biological regulation | 24 | 0.85 |
| 2 | locomotion | 6 | 0.21 |
| 2 | localization | 13 | 0.46 |
| 2 | reproductive process | 8 | 0.28 |
| 2 | homeostatic process | 4 | 0.14 |
| 2 | extracellular structure organization and biogenesis | 1 | 0.04 |
| 3 | multicellular organismal development | 28 | 0.99 |
| 3 | embryonic development | 15 | 0.53 |
| 3 | post-embryonic development | 20 | 0.71 |
| 3 | cellular metabolic process | 39 | 1.38 |
| 3 | regulation of biological process | 23 | 0.82 |
| 3 | behavior | 12 | 0.43 |
| 3 | primary metabolic process | 35 | 1.24 |
| 3 | sexual reproduction | 11 | 0.39 |
| 3 | macromolecule metabolic process | 29 | 1.03 |
| 3 | anatomical structure development | 11 | 0.39 |
| 3 | regulation of growth | 9 | 0.32 |
| 3 | establishment of localization | 13 | 0.46 |
| 3 | regulation of cellular process | 12 | 0.43 |
| 3 | cellular developmental process | 9 | 0.32 |
| 3 | regulation of locomotion | 4 | 0.14 |
| 3 | cell homeostasis | 3 | 0.11 |
| 3 | cell proliferation | 2 | 0.07 |
| 3 | cell communication | 5 | 0.18 |
| 3 | response to stress | 2 | 0.07 |
| 3 | reproductive developmental process | 6 | 0.21 |
| 3 | body growth | 3 | 0.11 |
| 3 | chemical homeostasis | 2 | 0.07 |
| 3 | excretion | 1 | 0.04 |
| 3 | cell cycle | 5 | 0.18 |
| 3 | catabolic process | 11 | 0.39 |
| 3 | response to chemical stimulus | 2 | 0.07 |
| 3 | immune system process | 2 | 0.07 |
| 3 | cell division | 1 | 0.04 |
| 3 | regulation of body fluids | 1 | 0.04 |
| 3 | chromosome segregation | 1 | 0.04 |
| 3 | cell organization and biogenesis | 5 | 0.18 |
| 3 | regulation of metabolic process | 5 | 0.18 |
| 3 | death | 4 | 0.14 |
| 3 | biosynthetic process | 4 | 0.14 |
| 3 | ensheathment of neurons | 1 | 0.04 |
| 3 | aging | 1 | 0.04 |
| 3 | intercellular bridge organization and biogenesis | 1 | 0.04 |
| 3 | response to endogenous stimulus | 1 | 0.04 |
| 3 | neurological process | 2 | 0.07 |
| 3 | protein localization | 1 | 0.04 |
| 3 | regulation of biological quality | 1 | 0.04 |
| 3 | regulation of multicellular organismal process | 1 | 0.04 |

b. Molecular function

| **GO Graph Level** | **GO Term** | **Representation** | **% Representation of total** |
| --- | --- | --- | --- |
| **1** | **molecular_function** | **77** | **2.73** |
| 2 | binding | 43 | 1.53 |
| 2 | catalytic activity | 45 | 1.60 |
| 2 | structural molecule activity | 7 | 0.25 |
| 2 | transporter activity | 5 | 0.18 |
| 2 | transcription regulator activity | 1 | 0.04 |
| 2 | antioxidant activity | 1 | 0.04 |
| 2 | signal transducer activity | 2 | 0.07 |
| 2 | enzyme regulator activity | 2 | 0.07 |
| 2 | auxiliary transport protein activity | 1 | 0.04 |
| 3 | nucleotide binding | 13 | 0.46 |
| 3 | protein binding | 7 | 0.25 |
| 3 | ion binding | 13 | 0.46 |
| 3 | oxidoreductase activity | 11 | 0.39 |
| 3 | transferase activity | 14 | 0.50 |
| 3 | hydrolase activity | 14 | 0.50 |
| 3 | nucleic acid binding | 6 | 0.21 |
| 3 | hormone binding | 1 | 0.04 |
| 3 | structural constituent of ribosome | 1 | 0.04 |
| 3 | lyase activity | 4 | 0.14 |
| 3 | receptor activity | 1 | 0.04 |
| 3 | structural constituent of eye lens | 1 | 0.04 |
| 3 | ion transporter activity | 3 | 0.11 |
| 3 | cofactor binding | 2 | 0.07 |
| 3 | receptor signaling protein activity | 1 | 0.04 |
| 3 | GTPase regulator activity | 1 | 0.04 |
| 3 | transcriptional elongation regulator activity | 1 | 0.04 |
| 3 | enzyme activator activity | 1 | 0.04 |
| 3 | RNA polymerase II transcription factor activity | 1 | 0.04 |
| 3 | channel regulator activity | 1 | 0.04 |
| 3 | tetrapyrrole binding | 1 | 0.04 |
| 3 | deaminase activity | 1 | 0.04 |
| 3 | vitamin binding | 1 | 0.04 |
| 3 | isomerase activity | 2 | 0.07 |
| 3 | phosphatase regulator activity | 1 | 0.04 |
| 3 | carrier activity | 1 | 0.04 |
| 3 | ligase activity | 1 | 0.04 |
| 3 | carbohydrate transporter activity | 1 | 0.04 |

**c. Cellular component**

| **GO Graph Level** | **GO Term** | **Representation** | **% Representation of total** |
| --- | --- | --- | --- |
| 1 | cellular_component | 45 | 1.60 |
| 2 | cell | 45 | 1.60 |
| 2 | protein complex | 11 | 0.39 |
| 2 | organelle | 28 | 0.99 |
| 2 | organelle part | 8 | 0.28 |
| 2 | extracellular region part | 2 | 0.07 |
| 2 | extracellular region | 2 | 0.07 |
| 2 | extracellular matrix part | 1 | 0.04 |
| 2 | envelope | 5 | 0.18 |
| 2 | extracellular matrix | 1 | 0.04 |
| 2 | membrane-enclosed lumen | 2 | 0.07 |
| 3 | cell part | 45 | 1.60 |
| 3 | membrane-bound organelle | 23 | 0.82 |
| 3 | non-membrane-bound organelle | 8 | 0.28 |
| 3 | extracellular space | 1 | 0.04 |
| 3 | organelle envelope | 5 | 0.18 |
| 3 | extracellular matrix (sensu Metazoa) | 1 | 0.04 |
| 3 | organelle lumen | 2 | 0.07 |
| 3 | vesicle | 1 | 0.04 |

**Female dataset**

**a. Biological process**

| **GO Graph Level** | **GO Term** | **Representation** | **% Representation of total** |
| --- | --- | --- | --- |
| **1** | **biological_process** | **88** | **2.60** |
| 2 | cellular process | 68 | 2.01 |
| 2 | multicellular organismal process | 34 | 1.00 |
| 2 | developmental process | 33 | 0.97 |
| 2 | metabolic process | 56 | 1.65 |
| 2 | growth | 9 | 0.27 |
| 2 | response to stimulus | 15 | 0.44 |
| 2 | biological regulation | 24 | 0.71 |
| 2 | reproduction | 10 | 0.29 |
| 2 | localization | 14 | 0.41 |
| 2 | homeostatic process | 4 | 0.12 |
| 2 | reproductive process | 6 | 0.18 |
| 2 | biological adhesion | 1 | 0.03 |
| 2 | multi-organism process | 1 | 0.03 |
| 2 | locomotion | 1 | 0.03 |
| 2 | maintenance of localization | 1 | 0.03 |
| 3 | multicellular organismal development | 31 | 0.91 |
| 3 | cellular metabolic process | 53 | 1.56 |
| 3 | embryonic development | 21 | 0.62 |
| 3 | primary metabolic process | 51 | 1.50 |
| 3 | macromolecule metabolic process | 48 | 1.42 |
| 3 | regulation of biological process | 23 | 0.68 |
| 3 | post-embryonic development | 13 | 0.38 |
| 3 | response to stress | 8 | 0.24 |
| 3 | aging | 15 | 0.44 |
| 3 | establishment of localization | 14 | 0.41 |
| 3 | regulation of cellular process | 18 | 0.53 |
| 3 | behavior | 6 | 0.18 |
| 3 | cell cycle | 6 | 0.18 |
| 3 | regulation of growth | 5 | 0.15 |
| 3 | chromosome segregation | 3 | 0.09 |
| 3 | cell organization and biogenesis | 14 | 0.41 |
| 3 | anatomical structure development | 9 | 0.27 |
| 3 | biosynthetic process | 15 | 0.44 |
| 3 | response to endogenous stimulus | 3 | 0.09 |
| 3 | body growth | 4 | 0.12 |
| 3 | cell proliferation | 2 | 0.06 |
| 3 | regulation of developmental process | 3 | 0.09 |
| 3 | sexual reproduction | 3 | 0.09 |
| 3 | cell homeostasis | 3 | 0.09 |
| 3 | chemical homeostasis | 3 | 0.09 |
| 3 | response to biotic stimulus | 2 | 0.06 |
| 3 | regulation of metabolic process | 12 | 0.35 |
| 3 | cell adhesion | 1 | 0.03 |
| 3 | response to chemical stimulus | 3 | 0.09 |
| 3 | reproductive developmental process | 4 | 0.12 |
| 3 | catabolic process | 6 | 0.18 |
| 3 | cell division | 2 | 0.06 |
| 3 | pollination | 1 | 0.03 |
| 3 | regulation of locomotion | 1 | 0.03 |
| 3 | molting cycle | 1 | 0.03 |
| 3 | cellular developmental process | 4 | 0.12 |
| 3 | protein localization | 2 | 0.06 |
| 3 | nitrogen compound metabolic process | 6 | 0.18 |
| 3 | response to abiotic stimulus | 3 | 0.09 |
| 3 | response to external stimulus | 3 | 0.09 |
| 3 | cytokinetic process | 1 | 0.03 |
| 3 | detection of stimulus | 2 | 0.06 |
| 3 | neurological process | 2 | 0.06 |
| 3 | regulation of a molecular function | 1 | 0.03 |
| 3 | cell communication | 1 | 0.03 |
| 3 | death | 1 | 0.03 |

**b.** Molecular function

| **GO Graph Level** | **GO Term** | **Representation** | **% Representation of total** |
| --- | --- | --- | --- |
| **1** | **molecular_function** | **83** | **2.45** |
| 2 | binding | 50 | 1.47 |
| 2 | catalytic activity | 51 | 1.50 |
| 2 | structural molecule activity | 10 | 0.29 |
| 2 | transporter activity | 6 | 0.18 |
| 2 | transcription regulator activity | 3 | 0.09 |
| 2 | nutrient reservoir activity | 2 | 0.06 |
| 2 | translation regulator activity | 2 | 0.06 |
| 3 | protein binding | 18 | 0.53 |
| 3 | nucleic acid binding | 16 | 0.47 |
| 3 | nucleotide binding | 21 | 0.62 |
| 3 | structural constituent of ribosome | 7 | 0.21 |
| 3 | transferase activity | 17 | 0.50 |
| 3 | ion binding | 14 | 0.41 |
| 3 | hydrolase activity | 18 | 0.53 |
| 3 | oxidoreductase activity | 6 | 0.18 |
| 3 | lipid transporter activity | 3 | 0.09 |
| 3 | helicase activity | 2 | 0.06 |
| 3 | lyase activity | 4 | 0.12 |
| 3 | small protein activating enzyme activity | 2 | 0.06 |
| 3 | virion binding | 1 | 0.03 |
| 3 | isomerase activity | 4 | 0.12 |
| 3 | chromatin binding | 1 | 0.03 |
| 3 | structural constituent of cytoskeleton | 1 | 0.03 |
| 3 | ion transporter activity | 1 | 0.03 |
| 3 | ribonucleoprotein binding | 1 | 0.03 |
| 3 | ligase activity | 3 | 0.09 |
| 3 | carbohydrate binding | 1 | 0.03 |
| 3 | carrier activity | 1 | 0.03 |

**c.** Cellular component

| **GO Graph Level** | **GO Term** | **Representation** | **% Representation of total** |
| --- | --- | --- | --- |
| **1** | **cellular_component** | **62** | **1.83** |
| 2 | protein complex | 22 | 0.65 |
| 2 | organelle | 40 | 1.18 |
| 2 | cell | 59 | 1.74 |
| 2 | organelle part | 21 | 0.62 |
| 2 | extracellular region | 3 | 0.09 |
| 2 | extracellular region part | 2 | 0.06 |
| 2 | membrane-enclosed lumen | 7 | 0.21 |
| 2 | synapse | 1 | 0.03 |
| 2 | envelope | 3 | 0.09 |
| 3 | cell part | 59 | 1.74 |
| 3 | membrane-bound organelle | 28 | 0.83 |
| 3 | non-membrane-bound organelle | 18 | 0.53 |
| 3 | extracellular space | 2 | 0.06 |
| 3 | organelle lumen | 7 | 0.21 |
| 3 | organelle envelope | 3 | 0.09 |
| 3 | unlocalized protein complex | 1 | 0.03 |
