# Supplementary material for: In silico analysis of expressed sequence tags from Trichostrongylus vitrinus (Nematoda): comparison of the automated ESTExplorer workflow platform with conventional database searches
Source: BMC Bioinformatics. 2008 Feb 13;9(Suppl 1):S10. doi: 10.1186/1471-2105-9-S1-S10 (PMC2259411; doi:10.1186/1471-2105-9-S1-S10)
Supplement: Additional file 3 — Metabolic pathways in Trichostrongylus vitrinus mapped by Kyoto Encyclopedia of Genes and Genomes (KEGG). [file 1471-2105-9-S1-S10-S3.doc]

**Additional File 3:** In *silico* analysis of expressed sequence tags (EST) from *Trichostrongylus vitrinus* (Nematoda): comparison of the automated ESTExplorer workflow platform with database searches.

Shivashankar H. Nagaraj, Robin B. Gasser, Alasdair J. Nisbet and Shoba Ranganathan

**Table S3: Metabolic pathways in *Trichostrongylus vitrinus* mapped by Kyoto Encyclopedia of Genes and Genomes (KEGG)**

| Serial Number | | KEGG Pathway | | | Sequence Count | | Enzyme Count | Sequence IDs | |  |
| --- | --- | --- | --- | --- | --- | --- | --- | --- | --- | --- |
| **Male sequences** | | | | | | | | | |  |
| 1 | | | Glycolysis / Gluconeogenesis | 9 | | 9 | | | TVm08_H01;Contig117,Contig38,Contig103,Contig24,TVm06_F02,TVm11_A05,Contig65,TVm11_F11; |  |
| 2 | | | Carbon fixation | 7 | | 7 | | | TVm08_H01;TVm10_B03,Contig127,TVm05_F02,TVm09_E06,TVm11_A05,Contig65 |  |
| 3 | | | Pyruvate metabolism | 7 | | 7 | | | TVm08_H01;TVm10_B03,Contig127,TVm05_F02,Contig123,TVm09_E06,TVm11_F11; |  |
| 4 | | | Proteasome | 6 | | 0 | | | Contig140,Contig34,TVm10_H01,TVm03_A09,Contig96,Contig42 |  |
| 5 | | | Streptomycin biosynthesis | 3 | | 3 | | | Contig117,Contig38,Contig24 |  |
| 6 | | | Fructose and mannose metabolism | 5 | | 5 | | | TVm11_A05,Contig65,Contig117,Contig38,Contig24 |  |
| 7 | | | Adipocytokine signaling pathway | 4 | | 2 | | | TVm11_D01,TVm02_H04;Contig130,Contig123 |  |
| 8 | | | Aminosugars metabolism | 3 | | 3 | | | Contig117,Contig38,Contig24 |  |
| 9 | | | Galactose metabolism | 3 | | 3 | | | Contig117,Contig38,Contig24 |  |
| 10 | | | Pentose phosphate pathway | 3 | | 3 | | | TVm11_A05,Contig65,Contig103 |  |
| 11 | | | Starch and sucrose metabolism | 4 | | 4 | | | Contig117,Contig38,Contig24,Contig103 |  |
| 12 | | | Signal transduction mechanisms | 5 | | 5 | | | TVm04_F01,TVm01_E10,TVm06_D01,TVm06_H04,TVm03_B10; |  |
| 13 | | | Amyotrophic lateral sclerosis (ALS) | 1 | | 0 | | | TVm06_E04 |  |
| 14 | | | Cell adhesion molecules (CAMs) | 1 | | 0 | | | Contig69 |  |
| 15 | | | Apoptosis | 2 | | 1 | | | TVm06_E04,Contig43 |  |
| 16 | | | Other enzymes | 8 | | 8 | | | Contig101,TVm09_B11,TVm06_C06,TVm07_H07,TVm07_D11,TVm08_H06,TVm05_C03,TVm06_A03 |  |
| 17 | | | Propanoate metabolism | 2 | | 2 | | | Contig51,TVm11_F11; |  |
| 18 | | | Major facilitator superfamily (MFS) | 1 | | 0 | | | Contig130 |  |
| 19 | | | Type II diabetes mellitus | 1 | | 1 | | | TVm08_H01; |  |
| 20 | | | B cell receptor signaling pathway | 1 | | 1 | | | Contig43 |  |
| 21 | | | Glyoxylate and dicarboxylate metabolism | 1 | | 1 | | | TVm09_E06 |  |
| 22 | | | Citrate cycle (TCA cycle) | 2 | | 2 | | | TVm09_E06,Contig123 |  |
| 23 | | | Inorganic ion transport and metabolism | 1 | | 1 | | | TVm11_H05 |  |
| 24 | | | Nicotinate and nicotinamide metabolism | 2 | | 2 | | | Contig53,TVm03_F08 |  |
| 25 | | | Fatty acid elongation in mitochondria | 1 | | 1 | | | Contig92 |  |
| 26 | | | Purine metabolism | 3 | | 3 | | | TVm08_H01;TVm04_B04,TVm02_H09 |  |
| 27 | | | Natural killer cell mediated cytotoxicity | 1 | | 1 | | | Contig43 |  |
| 28 | | | Reductive carboxylate cycle (CO2 fixation) | 1 | | 1 | | | TVm09_E06 |  |
| 29 | | | Valine, leucine and isoleucine degradation | 2 | | 2 | | | Contig51,Contig92 |  |
| 30 | | | Inositol phosphate metabolism | 2 | | 2 | | | Contig53,TVm03_F08 |  |
| 31 | | | Huntington's disease | 1 | | 0 | | | TVm06_E04 |  |
| 32 | | | Caprolactam degradation | 1 | | 1 | | | Contig92 |  |
| 33 | | | Benzoate degradation via CoA ligation | 2 | | 2 | | | Contig53,TVm03_F08 |  |
| 34 | | | Ubiquitin mediated proteolysis | 1 | | 1 | | | Contig114; |  |
| 35 | | | Cysteine metabolism | 1 | | 1 | | | TVm11_F11; |  |
| 36 | | | T cell receptor signaling pathway | 1 | | 1 | | | Contig43 |  |
| 37 | | | Axon guidance | 1 | | 1 | | | Contig43 |  |
| 38 | | | CD molecules | 1 | | 0 | | | Contig69 |  |
| 39 | | | Wnt signaling pathway | 2 | | 1 | | | TVm06_E04,Contig43 |  |
| 40 | | | Insulin signaling pathway | 2 | | 2 | | | TVm08_H01;Contig123 |  |
| 41 | | | Fatty acid metabolism | 2 | | 2 | | | TVm11_D01,Contig92 |  |
| 42 | | | MAPK signaling pathway | 2 | | 1 | | | TVm06_E04,Contig43 |  |
| 43 | | | Basal transcription factors | 1 | | 0 | | | TVm01_F11 |  |
| 44 | | | Glycerophospholipid metabolism | 1 | | 1 | | | TVm11_G05 |  |
| 45 | | | Long-term potentiation | 1 | | 1 | | | Contig43 |  |
| 46 | | | Butanoate metabolism | 1 | | 1 | | | Contig92 |  |
| 47 | | | Calcium signaling pathway | 1 | | 1 | | | Contig43 |  |
| 48 | | | Tryptophan metabolism | 1 | | 1 | | | Contig92 |  |
| 49 | | | Lysine degradation | 1 | | 1 | | | Contig92 |  |
| 50 | | | ATP synthesis | 1 | | 1 | | | TVm04_E02 |  |
| 51 | | | Cell cycle | 1 | | 0 | | | TVm06_E04 |  |
| 52 | | | Gap junction | 1 | | 0 | | | TVm07_C10; |  |
| 53 | | | Ribosome | 1 | | 0 | | | Contig52 |  |
|  | | | **Total for male sequences** | **120** | | **100** | | |  |  |
| **Female sequences** | | | | | | | | | | |
| 1 | Complement and coagulation cascades | | | 1 | | 1 | | | Contig28; | |
| 2 | Glycolysis / Gluconeogenesis | | | 8 | | 8 | | | Contig85;TVf03_B11,Contig100,Contig56;,Contig72,Contig77,Contig10,TVf10_C05 | |
| 3 | Pyruvate metabolism | | | 6 | | 6 | | | Contig85;,TVf03_B11,Contig108,Contig134;,Contig72,TVf10_C05 | |
| 4 | Ribosome | | | 9 | | 0 | | | Contig96;Contig125,Contig129,Contig103,TVf06_G10;,Contig70,TVf10_C10,TVf11_G05,Contig47 | |
| 5 | Valine, leucine and isoleucine biosynthesis | | | 3 | | 3 | | | Contig85;Contig72,TVf03_B11 | |
| 6 | Other ion-coupled transporters | | | 3 | | 1 | | | Contig66,Contig67,Contig25 | |
| 7 | Aminosugars metabolism | | | 3 | | 3 | | | TVf10_B11,TVf04_G02;,Contig69 | |
| 8 | Other replication, recombination and repair proteins | | | 2 | | 2 | | | Contig78,Contig130 | |
| 9 | Lysine biosynthesis | | | 1 | | 1 | | | Contig120 | |
| 10 | Biosynthesis of ansamycins | | | 1 | | 1 | | | TVf06_B03 | |
| 11 | Glycerolipid metabolism | | | 3 | | 3 | | | Contig134;,TVf06_H10;,Contig10 | |
| 12 | Antigen processing and presentation | | | 2 | | 0 | | | Contig119,TVf10_B04; | |
| 13 | Prion disease | | | 1 | | 0 | | | TVf06_G10; | |
| 14 | Amyotrophic lateral sclerosis (ALS) | | | 1 | | 1 | | | Contig120 | |
| 15 | Butanoate metabolism | | | 3 | | 3 | | | Contig85;Contig72,TVf03_B11 | |
| 16 | Carbon fixation | | | 2 | | 2 | | | TVf10_C05,TVf06_B03 | |
| 17 | Galactose metabolism | | | 2 | | 2 | | | Contig134;Contig77 | |
| 18 | Pentose phosphate pathway | | | 2 | | 2 | | | Contig77,TVf06_B03 | |
| 19 | Parkinson's disease | | | 1 | | 1 | | | TVf10_F06 | |
| 20 | Pores ion channels | | | 1 | | 0 | | | Contig119 | |
| 21 | Protein folding and associated processing | | | 3 | | 2 | | | TVf01_D12,TVf03_E07;,Contig119 | |
| 22 | Purine metabolism | | | 4 | | 4 | | | TVf03_A03,TVf10_C05,TVf02_E01,Contig6; | |
| 23 | Streptomycin biosynthesis | | | 1 | | 1 | | | Contig77 | |
| 24 | Type II diabetes mellitus | | | 1 | | 1 | | | TVf10_C05 | |
| 25 | Glyoxylate and dicarboxylate metabolism | | | 1 | | 1 | | | Contig108 | |
| 26 | Other amino acid metabolism | | | 2 | | 2 | | | Contig141,TVf05_G09; | |
| 27 | Glutamate metabolism | | | 2 | | 2 | | | TVf04_G02;Contig69 | |
| 28 | Gap junction | | | 3 | | 0 | | | Contig68;,TVf10_D12,TVf08_H01; | |
| 29 | Proteasome | | | 2 | | 2 | | | Contig104,Contig107 | |
| 30 | Phenylalanine, tyrosine and tryptophan biosynthesis | | | 1 | | 1 | | | Contig100 | |
| 31 | Glycerophospholipid metabolism | | | 2 | | 2 | | | TVf06_H10;TVf05_F08 | |
| 32 | Caprolactam degradation | | | 1 | | 1 | | | Contig10 | |
| 33 | Calcium signaling pathway | | | 2 | | 0 | | | Contig67,Contig25 | |
| 34 | Pentose and glucuronate interconversions | | | 1 | | 1 | | | Contig134; | |
| 35 | Ubiquitin mediated proteolysis | | | 1 | | 1 | | | TVf10_F06 | |
| 36 | Hedgehog signaling pathway | | | 1 | | 0 | | | Contig50 | |
| 37 | CD molecules | | | 1 | | 0 | | | Contig61 | |
| 38 | Cell cycle | | | 2 | | 0 | | | TVf06_A05,Contig138 | |
| 39 | Translation factors | | | 2 | | 0 | | | Contig17,TVf04_B07 | |
| 40 | RNA polymerase | | | 1 | | 1 | | | TVf01_C02 | |
| 41 | Insulin signaling pathway | | | 2 | | 1 | | | Contig70,TVf10_C05 | |
| 42 | Aminoacyl-tRNA biosynthesis | | | 1 | | 1 | | | Contig120 | |
| 43 | Fructose and mannose metabolism | | | 1 | | 1 | | | Contig134; | |
| 44 | MAPK signaling pathway | | | 2 | | 1 | | | Contig119,TVf10_C04; | |
| 45 | Glutathione metabolism | | | 1 | | 1 | | | TVf10_C04; | |
| 46 | Basal transcription factors | | | 1 | | 0 | | | Contig111; | |
| 47 | Pyrimidine metabolism | | | 1 | | 1 | | | TVf02_E01 | |
| 48 | Other translation proteins | | | 1 | | 1 | | | Contig106 | |
| 49 | Metabolism of xenobiotics by cytochrome P450 | | | 1 | | 1 | | | TVf10_C04; | |
| 50 | General function prediction only | | | 1 | | 0 | | | TVf05_B04; | |
| 51 | Phosphatidylinositol signaling system | | | 1 | | 1 | | | TVf06_H10; | |
| 52 | Signal transduction mechanisms | | | 2 | | 2 | | | TVf02_E09;Contig131 | |
| 53 | Starch and sucrose metabolism | | | 1 | | 1 | | | Contig77 | |
| 54 | Tight junction | | | 1 | | 1 | | | Contig93; | |
| 55 | ATP synthesis | | | 1 | | 1 | | | Contig44; | |
| 56 | GTP-binding proteins | | | 1 | | 0 | | | Contig45; | |
| 57 | Other enzymes | | | 2 | | 2 | | | TVf01_D12,TVf10_E10 | |
|  | **Total for female sequences** | | | **110** | | **78** | | |  | |
