# Supplementary material for: In silico analysis of expressed sequence tags from Trichostrongylus vitrinus (Nematoda): comparison of the automated ESTExplorer workflow platform with conventional database searches
Source: BMC Bioinformatics. 2008 Feb 13;9(Suppl 1):S10. doi: 10.1186/1471-2105-9-S1-S10 (PMC2259411; doi:10.1186/1471-2105-9-S1-S10)
Supplement: Additional file 4 — An example output for functional classification of ESTs generated using the Blast2GO component of ESTEXplorer's Phase II – Nucleotide-level Analysis. [file 1471-2105-9-S1-S10-S4.ppt]

## Slide 1
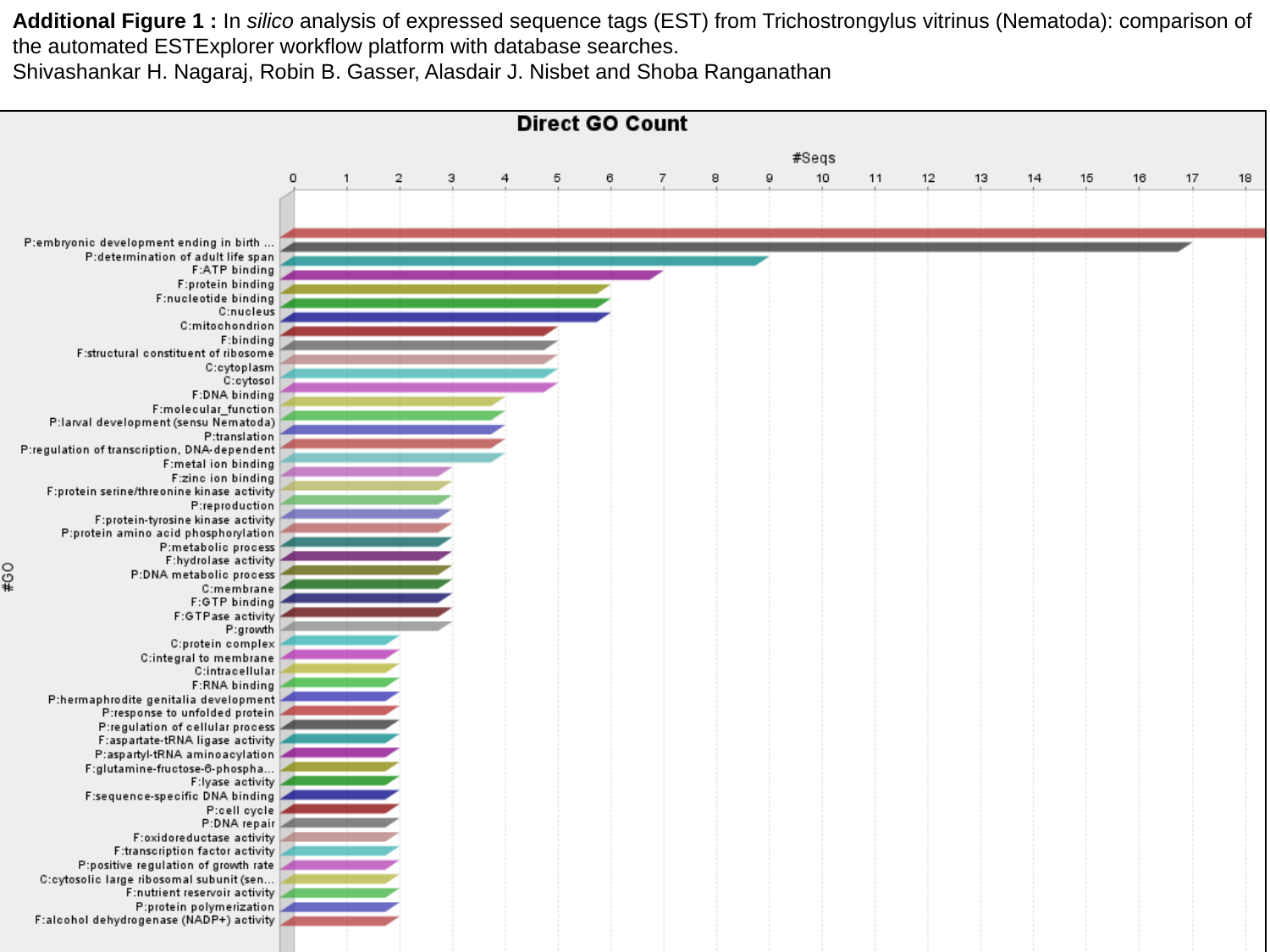

Additional Figure 1 : In silico analysis of expressed sequence tags (EST) from Trichostrongylus vitrinus (Nematoda): comparison of the automated ESTExplorer workflow platform with database searches.
Shivashankar H. Nagaraj, Robin B. Gasser, Alasdair J. Nisbet and Shoba Ranganathan
